# Ternary MOF-on-MOF heterostructures with controllable architectural and compositional complexity via multiple selective assembly

**DOI:** 10.1038/s41467-020-18776-z

**Published:** 2020-10-02

**Authors:** Chao Liu, Qiang Sun, Lina Lin, Jing Wang, Chaoqi Zhang, Chunhong Xia, Tong Bao, Jingjing Wan, Rong Huang, Jin Zou, Chengzhong Yu

**Affiliations:** 1 School of Chemistry and Molecular Engineering, East China Normal University, 200241 Shanghai, P. R. China; 2 Materials Engineering, University of Queensland, Brisbane, QLD 4072 Australia; 3 Key Laboratory of Polar Materials and Devices (MOE), Department of electronics, East China Normal University Shanghai, 200241 Shanghai, P. R. China; 4 Centre for Microscopy and Microanalysis, University of Queensland, Brisbane, QLD 4072 Australia; 5 Australian Institute for Bioengineering and Nanotechnology, The University of Queensland, Brisbane, QLD 4072 Australia

**Keywords:** Chemistry, Materials science, Nanoscience and technology

## Abstract

Assembly of different metal-organic framework (MOF) building blocks into hybrid MOF-on-MOF heterostructures is promising in chemistry and materials science, however the development of ternary MOF-on-MOF heterostructures with controllable architectural and compositional complexity is challenging. Here we report the synthesis of three types of ternary MOF-on-MOF heterostructures via a multiple selective assembly strategy. This strategy relies on the choice of one host MOF with more than one facet that can arrange the growth of a guest MOF, where the arrangement is site-selective without homogenous growth of guest MOF or homogenous coating of guest on host MOF. The growth of guest MOF on a selected site of host MOF in each step provides the opportunity to further vary the combinations of arrangements in multiple steps, leading to ternary MOF-on-MOF heterostructures with tunable complexity. The developed strategy paves the way towards the rational design of intricate and unprecedented MOF-based superstructures for various applications.

## Abstract (web summary)

Assembly of MOF-on-MOF hybrids with complex structures and properties is of interest, but achieving sophisticated ternary heterostructures is challenging. Here, the authors synthesize three types of ternary MOF-on-MOF heterostructures with tunable complexity via a multiple selective assembly strategy.

## Introduction

Hybrid materials especially nanostructures with high complexity have attracted intensive attention due to their elegant structures and elaborate functionalities^1–4^. The nanostructural complexity can be classified according to (1) composition (e.g., conjugating different components)^5,6^, (2) architecture (e.g., manipulating geometric arrangements)^7–9^, and (3) the integration of both compositional and architectural diversity into one nanostructrure^10–12^. As an important family of porous crystalline materials, the composition and architecture of metal-organic frameworks (MOFs) are both key factors for their properties and applications^13–19^. To improve the complexity of MOF-based composites with broadened functions, the assembly of MOFs and other nanomaterials is a promising approach^20–23^. Meanwhile, conjugation of two or more different MOFs into one MOF-on-MOF hybrid material presents another strategy for the fabrication of complicated MOF hybrids with unprecedented nanostructures^24–35^.

To date, various architectures of MOF-on-MOF hybrids including core-shell^24,27,36,37^, yolk-shell^25^, asymmetric structure^28,38,39^ and film on film^32,40^ have been developed. However, the present MOF-on-MOF systems are mainly focused on binary compositions. Construction of ternary MOF-on-MOF hybrids has limited success. Kitagawa and colleagues prepared a NH_2_-UIO-66&MOF-76@NH_2_-MIL-125 MOF-on-MOF hybrid with guest MOFs randomly adhered on host via a surfactant-assisted route^41^. A ternary MOF-on-MOF film by sequential epitaxial growth of three different Cu-based MOFs on Cu(OH)_2_ modified foil is demonstrated by Takashi and co-workers^42^. Very recently, a double shelled core-shell Fe-MIL-88B@Ga-MIL-88B@Fe-MIL-88C ternary MOF-on-MOF heterostructure with similar compositions is reported by Oh and colleagues^43^. To our knowledge, there is no report on the synthesis of ternary MOF-on-MOF materials with controllable architectural and compositional complexity.

**Fig. 1:**
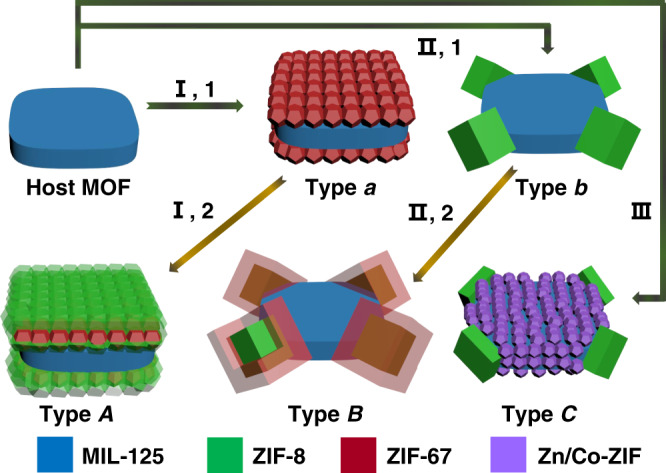
Illustration of the multiple selective synthesis approaches (I–III) and structures of ternary MOF-on-MOF heterostructures (types *A*–*C*). In approach **I**, ZIF-67 nanocrystals are selectively grown on top-bottom surfaces of MIL-125 in the first step (**I**, 1), forming a type *a* binary heterostructure. By further growth of ZIF-8 on ZIF-67 in *a* in the second step (**I**, 2), type *A* is synthesized. In approach **II**, a type *b* binary heterostructure is synthesized by growing ZIF-8 nanocrystals on the four corners of MIL-125 as an intermediate (**II**, 1). By further growth of ZIF-67 on ZIF-8 of *b* (**II**, 2), type *B* is generated. In approach **III**, Type *C* is obtained in one step by simultaneous growth of ZIF-8 on the four corners and Zn, Co-ZIF on the two top-bottom surfaces as well as four side surfaces.

Herein, we report the synthesis of three types of ternary MOF-on-MOF hybrids with adjustable architectural complexity and a distinct compositional combination consisted of three different MOFs as building blocks (Fig. 1). By using cake-like MIL-125 as the host MOF, ternary MOF-on-MOF hybrids, including quintuple sandwich-like (type *A*), cake@tetrapods with core-shell-structured pod (type *B*) and cake@tetrapods with core-shell-structured cake (type *C*) heterostructures, are obtained via a multiple selective assembly strategy in three approaches. In approaches **I** and **II**, the guest MOFs of ZIF-67 and ZIF-8 can be selectively grown on the two top-bottom surfaces (**I**, 1) and four corners (**II**, 1) of MIL-125 nanocakes in the first step, respectively, forming type *a* and *b* binary MOF-on-MOF heterostructures as seeds. The second-step site-selective growth of ZIF-8 (**I**, 2) and ZIF-67 (**II**, 2), respectively, on the ZIF-67 and ZIF-8 blocks in type *a* and *b* binary MOF-on-MOF seeds results in type A and type B ternary MOF-on-MOF heterostructures. For type *C* ternary MOF-on-MOF heterostructure, ZIF-8 pods are adhered on the four corners of core-shell-structured cake, which is formed by Zn, Co-ZIF shell coated on the two top-bottom and four side surfaces of MIL-125 core in a one-step approach (**III**).

## Results

### Host MOFs: cake-like MIL-125

First, a tetragonal structured Ti-centered MOF (MIL-125, space group of *I*4/mmm with the lattice parameters of *a* = 18.65 Å and *c* = 18.14 Å) was synthesized as the host via a solvothermal method^44^. The field-emission scanning electron microscopy (SEM) images (Supplementary Fig. 1a, b) of MIL-125 show highly dispersed nanoparticles with a cake-like morphology and smooth surface. The length and thickness are estimated to be 500–640 and 125 nm, respectively. Transmission electron microscopy (TEM) images (Supplementary Fig. 1c, d) show the solid nature of MIL-125. The crystallinity of MIL-125 is confirmed by X-ray diffraction (XRD) pattern (Supplementary Fig. 2), which is in accordance with the simulated result. By using MIL-125 nanocakes as host MOFs, three different ternary MOF-on-MOF heterostructures are synthesized as discussed below.

### Type *A* ternary MOF-on-MOF heterostructure

**Fig. 2:**
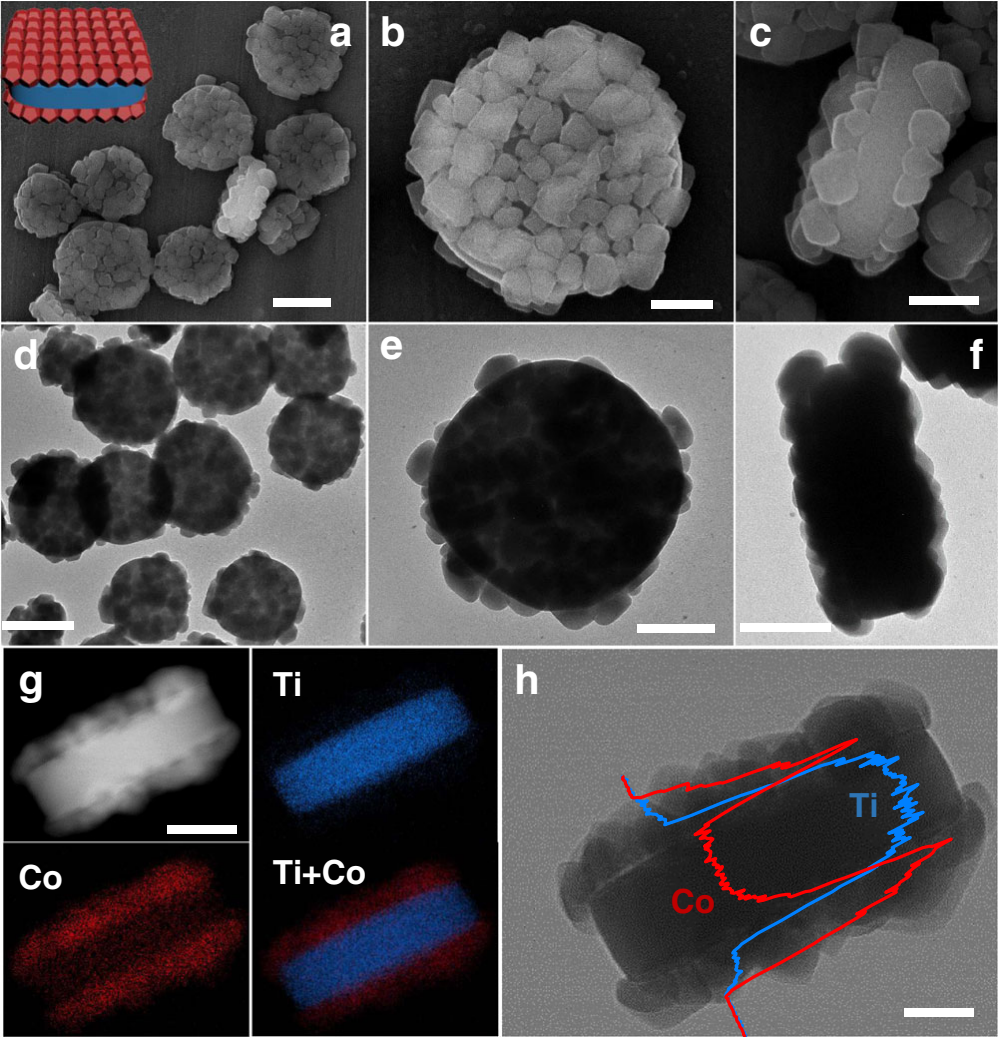
Type *a* binary heterostructures. **a**–**c** SEM images, **d**–**f** TEM images, **g** STEM and corresponding element mapping images, (**h**) TEM image and line scanning spectra of type *a* binary heterostructure. Scale bars are 500 nm (**a**, **d**), 200 nm (**b**, **c**, **e**–**g**), and (**h**) 100 nm, respectively. Inset of **a** is the structural illustration of type *a*.

By directly immersing MIL-125 nanocakes into the 2-methylimidazole (2-MeIM) and Co^2+^ methanol solution, MIL-125@ZIF-67 type *a* binary heterostructure was synthesized. The XRD pattern of type *a* binary hybrid shows the co-existence of diffraction peaks of both ZIF-67 and MIL-125 (Supplementary Fig. 3). SEM image (Fig. 2a) shows a well-dispersed cake-like morphology with a particle size of ~620–700 nm and rough surface. In the enlarged SEM images (Fig. 2b, c), ZIF-67 nanocrystals with diameters of ~50–100 nm are observed and selectively adhered on the two top-bottom surfaces of MIL-125 rather than on the corners or side surfaces, generating a triple layered sandwich-like ZIF-67@MIL-125@ZIF-67 superstructure. Some gaps between ZIF-67 nanocrystals are also observed. The thickness of the sandwich-like structure is measured to be ~250 nm. TEM observations (Fig. 2d–f) further demonstrate that the type *a* binary heterostructure has a sandwich-like structure, in agreement with the SEM results. The high-angle annular dark field scanning TEM (HAADF-STEM) and corresponding element mapping images (Fig. 2g) viewed parallel to the nanocake top/bottom surface show that the middle Ti-rich layer is sandwiched by two side Co-rich layers, consistent with the energy dispersive X-ray spectroscopy (EDX) line scanning results (Fig. 2h).

**Fig. 3:**
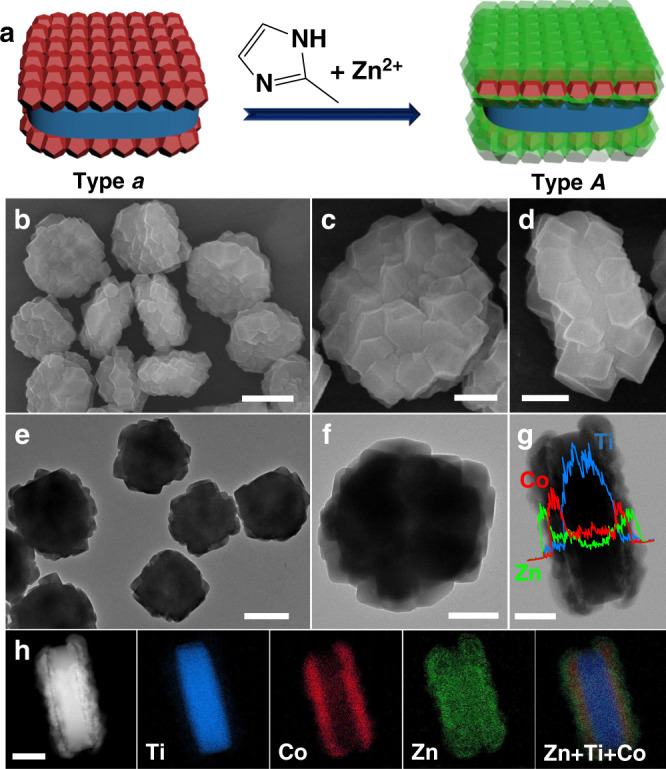
Type *A* ternary heterostructure. **a** Schematic illustration of the synthesis of type *A* ternary MOF-on-MOF heterostructure. **b**–**d** SEM images, **e**, **f** TEM images, **g** TEM image and corresponding line scanning spectra, **h** STEM image and corresponding element mapping images of type *A* ternary heterostructure. The scale bars are 500 nm (**b**, **d**) and 200 nm (**c**, **d**, **f**–**h**), respectively.

Using type *a* binary heterostructure as seeds for reaction with 2-MeIM and Zn^2+^, two extra ZIF-8 layers are selectively grown on ZIF-67 surfaces, forming type *A* ternary MOF-on-MOF heterostructure (Fig. 3a). The sandwich-like morphology is well kept as evidenced by both SEM (Fig. 3b–d) and TEM observations (Fig. 3e–g). The average diameters of nanocrystals coated on top/bottom surfaces increase to 100–200 nm (Fig. 3c), which are more densely packed compared to type *a* binary heterostructure. The thickness is increased to ~400 nm (Fig. 3d).

The co-existence of Zn, Co and Ti elements in type *A* heterostructure is demonstrated in the EDX spectrum (Supplementary Fig. 4). The line scanning spectra of a “vertical sandwich” (Fig. 3g) show the linear distribution of Zn, Co, and Ti, indicating that the inner Ti-rich layer (MIL-125) is sandwiched by two middle Co-rich layers (ZIF-67) and two outmost Zn-rich layers (ZIF-8), consistent with STEM and element mapping results showing a quintuple sandwich-like structure (Fig. 3h). The XRD pattern of the type *A* ternary heterostructure is shown in Supplementary Fig. 5. Although ZIF-8 and ZIF-67 with similar crystalline parameters can not be differentiated^45^, the relative intensity of the peaks assigned to ZIF versus MIL-125 is enhanced than that observed in type *a* binnary heterostructure, indicating the further growth of ZIF-8 on ZIF-67.

### Type *B* ternary MOF-on-MOF heterostructure

Type *b* binary heterostructure (MIL-125@ZIF-8) was synthesized according to our recent publication^46^. The SEM and TEM images (Supplementary Fig. 6a–e) display a unique MOF-on-MOF structure with ZIF-8 nanocrystals adhered on the four corner surfaces of MIL-125, generating a cake@tetrapods-like structure. The diameter of ZIF-8 is determined to be ~280 nm. The STEM image, line scanning profile and element mapping images (Supplementary Fig. 5f) show that the four Zn-rich faceted nanoparticles are preferentially formed on the corners of Ti-rich MIL-125 host. The detection of Zn in the middle region of MIL-125 may be due to the adsorption of positive Zn^2+^ by negatively charged MIL-125. The XRD pattern (Supplementary Fig. 7) also confirms the generation of crystalline ZIF-8 on MIL-125, in accordance with literature results.

**Fig. 4:**
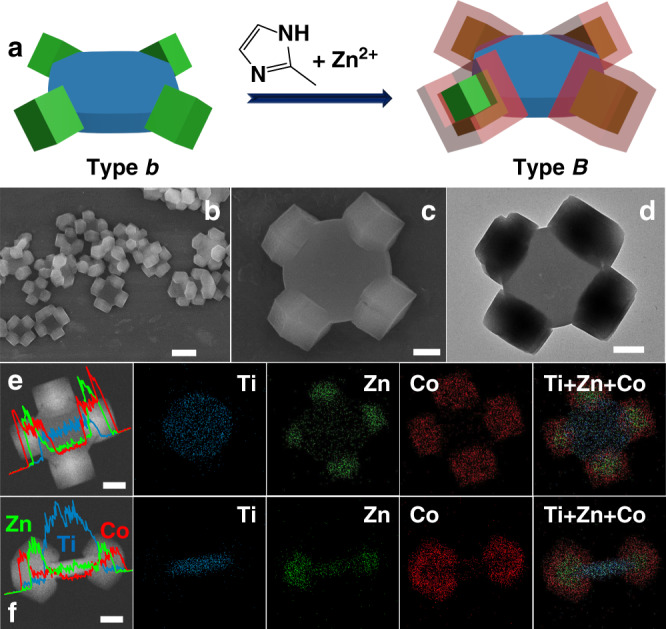
Type *B* ternary heterostructure. **a** Schematic illustration of the synthesis of type *B* ternary MOF-on-MOF heterostructure. **b**, **c** SEM images, **d** TEM images, **e**, **f** STEM images, line scanning spectra and corresponding element mapping images of type *B* ternary heterostructure. The scale bars are 500 nm (**b**) and 200 nm (**c**–**f**), respectively.

By further growth of ZIF-67 on type *b* binary heterostructure as the seeds (Fig. 4a), type *B* ternary MOF-on-MOF heterostructure is generated. SEM results show a similar morphology to type *b* binary heterostructure (Fig. 4b). The diameter of corner grown nanocrystals is measured to be ~500 nm in both SEM and TEM images (Fig. 4c, d), larger than the particle size of ZIF-8 in type *b* binary hybrid. The existence of Zn, Co and Ti elements in type *B* binary heterostructure is observed in the EDX spectrum (Supplementary Fig. 8). STEM image and line scanning spectra of Ti, Zn and Co elements are shown in Fig. 4e, f (view direction perpendicular and parallel to the top/bottom cake surface, respectively). Strong signal of Co and Zn is detected in the outer shell and inner core of pod part, respectively, while the cake body is abundant in Ti element. The superimposed images clearly display a cake@tetrapods-like structure with core-shell-structured (ZIF-8@ZIF-67) pods on the four corners of MIL-125 cake. The increased XRD peak intensity of ZIF further supports the growth of ZIF-67 on ZIF-8 (Supplementary Fig. 9), in accordance with SEM and TEM observations.

### Type *C* ternary MOF-on-MOF heterostructure

**Fig. 5:**
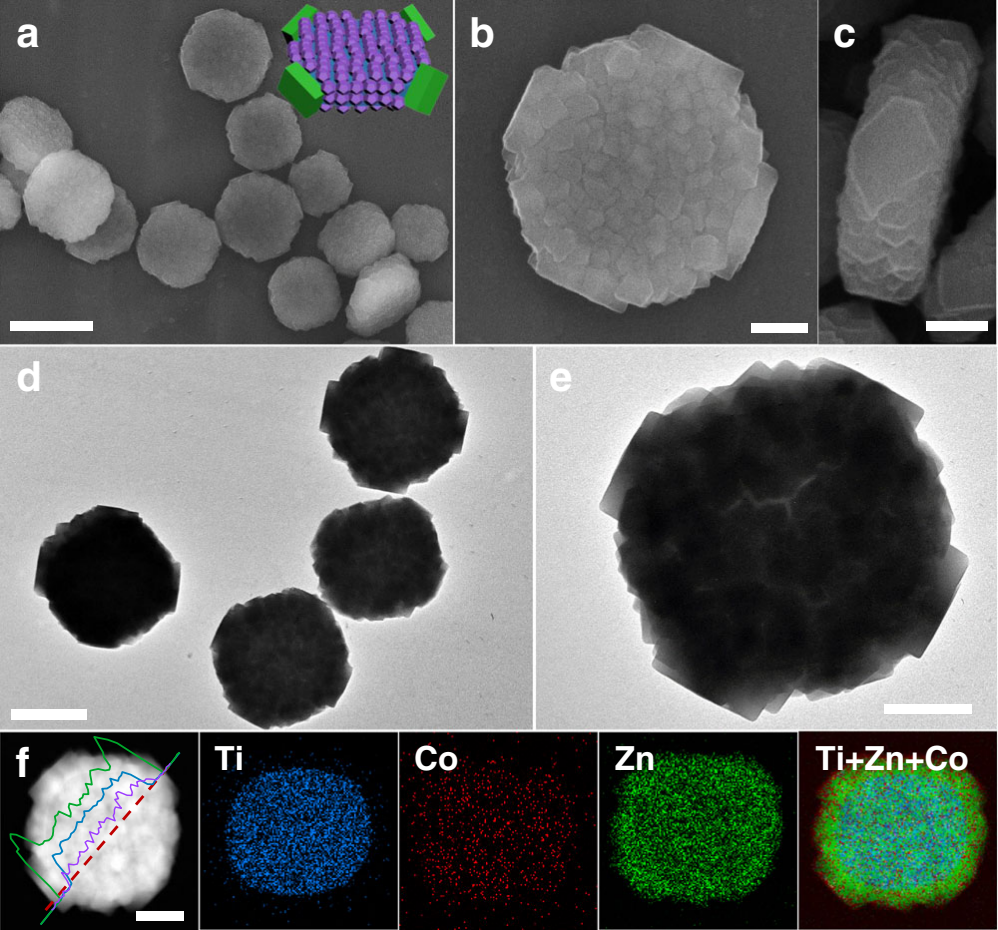
Type *C* ternary heterostructure. **a**–**c** SEM images, **d**, **e** TEM images, **f** STEM images, line scanning spectra and corresponding element mapping images of type *C* ternary MOF-on-MOF heterostructure. The scale bars are 1 μm (**a**), 500 nm (**d**) and 200 nm (**b**, **c**, **e**, **f**), respectively. Inset of **a** is the structural illustration of type *C*.

Zn^2+^ and Co^2+^ ions with a molar ratio of 7.5/2.5 together with 2-MeIM were used as precursors for growing guest ZIF on MIL-125 host to synthesize type *C* ternary heterostructure via a one-step reaction. Typical SEM and TEM images (Fig. 5a, d) show a uniform cake@tetrapods-like morphology with a rough surface in the cake body. The magnified images (Fig. 5b, e) display that larger nanocrystals (~150 nm) are attached on the four corners, while relatively smaller particles (~30 nm) are anchored on the top-bottom surface of MIL-125. Moreover, the small nanoparticles are also observed on the side surfaces in the image of a “vertical cake” (Fig. 5c).

The elements of Zn, Co, and Ti are simultaneously detected in the EDX spectrum of type *C* ternary heterostructure (Supplementary Fig. 10). The distribution of these elements is presented in the STEM image and corresponding line scanning spectra (Fig. 5f). The results show the distribution of Zn throughout the whole particle, while Co and Ti only distributed in the cake body, but not in the corner pod region. Moreover, the spot scan profile (Supplementary Fig. 11) also shows that only Zn is detected in the corner pod. The Zn/Co ratio detected in the cake body region was measured to be ~9/1. The XRD pattern of type *C* also shows the diffraction peaks of ZIF and MIL-125 (Supplementary Fig. 12). Thus, type *C* heterostructure is formed by the selective adhesion of ZIF-8 pods on the four corners of core-shell-structured cake, which is formed by Zn/Co-ZIF shell coated on the two top-bottom and four side surfaces of MIL-125 core.

Furthermore, bimetal precursor with Zn^2+^/Co^2+^ ratio of 0.75/0.25 was used to replace the single ones (Zn^2+^ or Co^2+^) for the secondary growth with type *a* and *b* as host, generating type *A*-1 and *B*-1 heterostructures. As evidenced by SEM and TEM images as well as XRD patterns and EDX spectra (Supplementary Figs. S13–18), type *A*-1 and *B*-1 heterostructures exhibit similar structures with type *A* and *B* with Zn/Co ratio changing from 3.2/1 and 1/2.1 to 2.8/1, and 6.3/1, respectively, suggesting the Zn/Co ratios in type *A* and *B* hybrids are tunable. In addition, the SEM and TEM images of three ternary MOF-on-MOF heterostructures after ultrasound treatment were also presented (Supplementary Figs. 19–21). No obvious changes were observed in type *A*, *B*, and *C* heterostructures, indicating good structural stability.

### Formation mechanism

To understand the formation mechanism of type *A*, *B* and *C* ternary heterostructures, the growth behavior of guest ZIFs on host MIL-125 was investigated. In the absence of MIL-125, the homogeneous nucleation of different ZIFs generates ZIF-67, ZIF-8, and Zn, Co-ZIF (Supplementary Fig. 22), consistent with reported results^47^. In the presence of MIL-125, the heterogenous nucleation of ZIF-8 on MIL-125 is driven by the interaction between exposed Ti-O cluster on four {110} facets of MIL-125 (i.e., the four corners) and linkers on the {001} facets of ZIF-8 according to our previous findings (Supplementary Figs. 23–25, and 26b)^46^. The exposed Ti-O clusters could be fully utilized to connect with 2-MeIM, leaving one Zn^2+^ not connected to the Ti-O node^46^. Further epitaxial growth of ZIF-8 on the corner of MIL-125, due to the lattice match of {110}_MIL-125_/{001}_ZIF-8_, generates type *b* heterostructure with slight lattice expansion of ZIF-8 (Supplementary Fig. 27).

It is also reported that ZIF-8 and ZIF-67 have isoreticular structures with similar unit cell parameters (ZIF-8, *a* = 16.99 Å; ZIF-67, *a* = 16.96 Å)^45^. The selective growth of ZIF-67 on the {001} facets of MIL-125, rather than ZIF-8 on the {110} facets, is worth of further investigation. The enlarged XRD patterns (Supplementary Fig. 28) of type *a* binary heterostructure show no shift of diffraction peaks of ZIF-67 or MIL-125, indicating the growth of ZIF-67 on MIL-125 is not epitaxial growth. When the growth time of ZIF-67 on MIL-125 was 2 min, SEM observations showed nucleates preferentially on the top-bottom surfaces of MIL-125 (Supplementary Fig. 29a). The nucleate sizes of ZIF-67 are obviously larger than ZIF-8 obtained at similar conditions (Supplementary Fig. 23), indicating faster nucleation of ZIF-67, possibly because a higher 2MeIM:Co^2+^ molar ratio (16:3) was used than that in the growth of type *b* heterostructure (2MeIM:Zn^2+^ molar ratio of 3:5, see details in Methods). The faster nucleation of ZIF-67 is further supported by the faster individual growth of ZIF-67 than ZIF-8 (Supplementary Fig. 30). The XRD pattern further demonstrates the occurrence of ZIF-67 nucleates (Supplementary Fig. 29b). Different from ZIF-8 in type *b* heterostructure, negligible peak shift is observed in the enlarged XRD pattern (Supplementary Fig. 29c), further suggesting the growth of type *a* heterostructure is not through epitaxial growth. By adding Co^2+^ first and then 2-MeIM to react with MIL-125 (Supplementary Fig. 31) or adding MIL-125 after homogenous nucleation of ZIF-67 clusters for 3 min (Supplementary Fig. 32), the generated materials were similar to type *a* binary heterostructure. Collectively, it is inferred that the growth of ZIF-67 goes through a homogenous nucleation stage in the first step (Supplementary Fig. 26a) due to the relatively fast nucleation rate.

To further understand why the generated ZIF-67 nucleates are selectively adhered on the {001} faces of MIL-125, the zeta potential of MIL-125 was measured to be −13.9 mV (Supplementary Fig. 33). To distinguish the surface charge difference at different facets of MIL-125, hexadecyl trimethyl ammonium bromide (CTAB, one cationic surfactant) was added during the growth of type *a* and *b* heterostructures. The zeta potential of MIL-125 was slightly increased with the addition of CTAB (Supplementary Fig. 33), indicating the adsorption of CTA^+^ onto negatively charged MIL-125 surface. The introduction of CTAB showed little influence on the formation of type *a* heterostructure, but the growth of individual ZIF-8 particles was observed in the synthesis system of type *b* heterostructure (Supplementary Figs. 34 and 35). The above results indicate that the positively charged CTA^+^ are preferentially absorbed on {110} rather than {001} facets of MIL-125, thus the growth of ZIF-8 is hindered while ZIF-67 can still grow on the top-down surfaces of MIL-125. These observations further suggest that the {110} facets of MIL-125 exhibit a lower surface charge than {001} facets, in accordance with literature reports that {110} facets of MIL-125 exposed a higher density of Ti-O clusters than the {001} facets^48^. In addition, the negatively charged nature of ZIF-67 is also confirmed by zeta potential analysis (Supplementary Fig. 36). It is suggested that ZIF-67 nucleates preferentially adhere on {001} rather than {110} facets of MIL-125 with relatively weaker electrostatic repulsion to reduce the surface free energy. Eventually type *a* binary heterostructure is formed.

As reported previously^42^, different ZIFs {[M(2-MeIM)_2_]_n_, M = Zn, Co or bimetal Zn/Co} exhibit isoreticular structures and almost the same unit cell parameters. Thus, epitaxial growth between different ZIFs is easily achieved due to their high lattice match degree of ~100%^21^. However, the growth of ZIFs on MIL-125 has a relatively lower degree of lattice match, e.g., ~97.6% between (001)_ZIF-8_/(110)_MIL-125_ facets. It is suggested that the better lattice match between ZIFs than ZIFs/MIL-125 results in the selective growth of ZIFs (ZIF-8, ZIF-67 or Zn, Co-ZIF) on ZIF-8 and ZIF-67 in type *a* and *b*, generating type *A* and *B* ternary MOF-on-MOF heterostructures.

When adding Co^2+^, Zn^2+^, and 2-MeIM together to react with MIL-125, the nucleation of ZIF-8 on the corner and Zn/Co-ZIF on top-bottom as well as side surfaces of MIL-125 possibly occurred simultaneously and could not be clearly differentiated (Supplementary Fig. 37). Smaller shift of ZIFs peaks is found in the enlarged XRD pattern of type *C* structure (Supplementary Fig. 38) compared to type *b*, due to the relatively lower amount of ZIF-8. When adding MIL-125 after reaction of Zn^2+^, Co^2+^, and 2-MeIM for 3 min, only small crystals were found on in some MIL-125 particles, the formation of relatively large sized ZIF-8 on the corner was not evident (Supplementary Fig. 39). It is suggested that in this approach (Supplementary Fig. 26c), the growth of ZIF-8 on corner of MIL-125 is through heterogeneous nucleation of ZIF-8 clusters on {110} facets of MIL-125, similar to type *b* hybrids. Simultaneously, the Zn/Co-ZIF clusters formed through homogenous nucleation adhered on the side ({100} facets) and top-bottom ({001} facets) surfaces of MIL-125. Consequently, type *C* ternary MOF-on-MOF heterostructures are formed.

It is noted that the growth of guest MOF on one specific facet of host MOF has been reported to generate binary MOF-on-MOF structures^28,37–39,43,46^. However, utilizing one host MOF with different facets that can individually regulate the growth of other guest MOF building blocks is demonstrated in this work. The successful synthesis of three types ternary MOF-on-MOF heterostructures relies on the choice of suitable host and guest MOF building blocks and understanding their interactions in the assembly process. In our synthesis system, mainly three types of MOF are used as building blocks. To enable the assembly of ternary MOF-on-MOF heterostructures with multiple complexity, three elements are important. (1) A host is chosen with more than one facet that can arrange the growth of a guest MOF. (2) The synthesis conditions should be adjusted to allow for a site-selective arrangement of MOF-on-MOF in each step. It is noted that homogenous growth of guest MOF (e.g., the formation of ZIF-8 mixed with MIL-125, Supplementary Fig. 25) or homogenous coating of guest MOF on host MOF should be avoided, because this would reduce the complexity of assembled structures. The site-selective growth of guest-host MOF-on-MOF can be driven by either heterogeneous nucleation of guest MOF on host followed by epitaxial growth (e.g., in type *b* binary hybrid), or homogeneous nucleation followed by electrostatic interaction (e.g., in type *a* binary hybrid). Nevertheless, the isoreticular growth is not recommended in the first step growth of MOF-on-MOF, but applied only in the second step of approaches **I** and **II** to grow guest MOF site-specifically on the binary hybrids. (3) Finally, by varying the combinations of guest-host arrangements in the multiple selective assembly strategy, ternary MOF-on-MOF heterostructure with multiple complexity can be obtained. Even approach **III** is a one-step synthesis, it is likely the growth of two guest MOF blocks on their preferred sites can be controlled by tuning the competition between two different guest-host MOF interactions (through optimizing the Co^2+^ and Zn^2+^ ratio, Supplementary Figs. S40 and 41), so that type *C* ternary MOF-on-MOF heterostructure can be successfully synthesized.

### Catalytic performance

To demonstrate the advantage of ternary MOF-on-MOF heterostructures, a photo-assisted peroxymonosulfate (PMS) activation system was established for degradation of methylene blue (MB, a typical dye pollutant) by using type *B* heterostructure as a catalyst (Supplementary Fig. S42a). For comparison, the catalytic performances of binary heterostructure (type *b*) and single MOFs (ZIF-8, ZIF-67 and MIL-125) were also tested. As shown in Supplementary Fig. 42b, 59.2% of MB was removed in 20 min by type *B* in the dark. Notably, under visible light irradiation, MB was totally removed within 5 min by type *B* heterostructure. For comparison, ZIF-8, ZIF-67 MIL-125 and type *b* heterostructure exhibited lower removal rates of 22.3, 75.0, 84.5, and 92.1% in 5 min. The enhanced performance type *B* ternary heterostructure over other samples is also supported by the total organic carbon (TOC) removal tests (Supplementary Fig. 43).

Furthermore, electron paramagnetic resonance (EPR) technique was employed to characterize the active radicals in the catalytic reaction systems. As illustrated in Supplementary Fig. 44a, EPR signals corresponding to •OH and SO_4_^•−^ were detected in all samples^49^. The intensity of these two radicals for type *B* heterostructure under irradiation is higher than that of type *B* in dark, ZIF-8, MIL-125, ZIF-67 and type *b* under irradiation, indicating that the activation of PMS is enhanced by the photocatalytic property of type *B* catalyst. Meanwhile, characteristic signal of O_2_^•−^ was also detected in the systems of type *B*, ZIF-8, MIL-125, ZIF-67 and type *b* under irradiation^44,46^, but not in type *B* in dark (Supplementary Fig. 44b).

The above results suggest that the improved activity of type *B* heterostructure may be correlated to the synergies of different building blocks (Supplementary Fig. 42a). Firstly, MIL-125 and ZIF-67 containing transitional metal centers with variable valences can activate PMS to generate •OH and SO_4_^•−^^50^. Moreover, ZIF-8/MIL-125 heterojunction^44,51^ improves the charge carrier separation as evidenced by the photocurrent characterization (Supplementary Fig. 45). The generated photoelectrons accelerate the activation of PMS for the production of •OH and SO_4_^•−^ radicals, which can also simultaneously reduce the O_2_ to yield O_2_^•−^ radicals. The improved photocatalytic performance of the ternary versus binary heterostructure is likely attributed to the further hybridization of ZIF-67 with higher activity for PMS activation than ZIF-8. Taking together, the synergistic effects, including chemical activation by both ZIF-67 and MIL-125, and photocatalytic activation by ZIF-8/MIL-125 heterojunction, lead to the enhanced photocatalytic performance of type *B* ternary heterostructure.

## Discussion

In conclusion, we have demonstrated a multiple selective assembly strategy for the synthesis of three distinctive types of ternary MOF-on-MOF hybrids with controllable architectural and compositional complexity. The choice of suitable host-guest MOFs and controlling the host-guest interaction allow for selective growth of MOF-on-MOF in each step, eventually ternary MOF-on-MOF heterostructures with high complexity are prepared in the multiple selective assembly process. Benefiting from the structural and compositional complexity, the resultant ternary heterostructures exhibit enhanced catalytic performance compared to binary hybrids and single MOF blocks. Our contributions are expected to provide guidance for design of MOF-based hybrid materials with sophisticated superstructures.

## Methods

### Chemicals

Titanium isopropoxide (TPOT, 97%, Aldrich), aminoterephthalate (99%, Aldrich), both zinc nitrate hexahydrate (Zn(NO_3_)_2_⋅6H_2_O, 98%) and cobalt nitrate hexahydrate (Co(NO_3_)_2_⋅6H_2_O) from Sinopharm Chemical Regent Co., Ltd, 2-methylimidazole (2-MeIM, Aldrich), N,N-dimethylformamide (DMF, 99%, Greagent) and methanol (AR, 99%, Adamas-beta) used as received. Millipore water was used in all experiments.

### Synthesis of MIL-125

The cake-like MIL-125 nanoparticles were prepared as follows. Typically, 0.6 mL of TPOT and 0.56 g of aminoterephthalate were dissolved into 40 mL mixture of DMF and CH_3_OH (9:1, v/v). The mixture was stirred for 5 min, then transferred to a 100 mL Teflon-line autoclave and heated at 150 °C for 24 h. Afterwards, the yellow solid product was collected by washing with DMF and methanol for three times. Finally, the as-synthesized MIL-125 was dispersed in 30 mL methanol to make a stock solution (~20 mg/mL) for further use.

### Synthesis of type *a*: MIL-125@ZIF-67 heterostructure

To synthesize type *a* heterostructure, 0.3 mL of MIL-125 stock solution (~ 6 mg of MIL-125), 5 mL of 80 mM 2-MeIM solution and 3 mL of 25 mM Co(NO_3_)_2_⋅6H_2_O solution were mixed under stirring. The mixture was allowed to react at room temperature for 4 h. The final product was collected by centrifugation, washed with methanol three times, and dispersed in 0.5 mL methanol (~36 mg/mL) for further use.

### Synthesis of type *A*: MIL-125@ZIF-67@ZIF-8 heterostructure

To synthesize type *A* heterostructure, 0.2 mL of the obtained type *a* dispersion, 3 mL of 25 mM 2-MeIM solution and 5 mL of 25 mM Zn(NO_3_)_2_⋅6H_2_O solution were mixed under stirring and then allowed to react at room temperature for 4 h. The product is then collected by centrifugation, washed with methanol for three times, and dried overnight.

### Synthesis of type *b*: MIL-125@ZIF-8 heterostructure

To synthesize MIL-125@ZIF-8 heterostructure, 0.4 mL of MIL-125 suspension (~8 mg of MIL-125), 6 mL of 25 mM 2-MeIM solution and 10 mL of 25 mM Zn(NO_3_)_2_⋅6H_2_O solution were mixed and then allowed to react at room temperature for 4 h. The product was collected by centrifugation, washed with methanol for three times, then dispersed in 0.4 mL methanol for further use.

### Synthesis of type *B*: MIL-125@ZIF-8@ZIF-67 heterostructure

To synthesize type *B* heterostructure, 0.4 mL of the obtained type *b* solution, 5 mL of 80 mM 2-MeIM solution and 3 mL of 25 mM Co(NO_3_)_2_⋅6H_2_O solution were mixed and then allowed to react at room temperature for 4 h. The product was then collected by centrifugation, washed with methanol several times, and dried overnight.

### Synthesis of type *C*: MIL-125@ZIF-8/Zn, Co-ZIF heterostructure

To synthesize type *C* heterostructure, 0.2 mL of MIL-125 suspension, 5 mL of 50 mM 2-MeIM solution and 3 mL of 25 mM Zn(NO_3_)_2_⋅6H_2_O/Co(NO_3_)_2_⋅6H_2_O (Zn/Co molar ratio of 7.5/2.5) solution were mixed and then allowed to react at room temperature for 4 h. The product was then collected by centrifugation, washed with methanol three times, and dried overnight.

### Synthesis of type *A*-1 MIL-125@ZIF-67@Zn/Co-ZIF heterostructure

The synthetic process is similar to that of type *A*, except using 5 mL of 50 mM 2-MeIM solution and 3 mL of 25 mM Zn(NO_3_)_2_⋅6H_2_O/Co(NO_3_)_2_⋅6H_2_O (Zn/Co molar ratio of 7.5/2.5) as precursor.

### Synthesis of type *B*-1 MIL-125@ZIF-8@Zn/Co-ZIF heterostructure

The synthetic process is similar to that of type *B*, except using 5 mL of 50 mM 2-MeIM solution and 3 mL of 25 mM Zn(NO_3_)_2_⋅6H_2_O/Co(NO_3_)_2_⋅6H_2_O (Zn/Co molar ratio of 7.5/2.5) as precursor.

### Material characterization

TEM images were obtained with Hitachi HT7700 at 120 KV. Chemical composition analyses were carried out using a JEM-2100F (JEOL, Japan) operating at 200 kV equipped with an X-ray energy dispersive spectrometer (EDS: X-Max 80 T, Oxford, UK). XRD patterns were recorded using a Bruker D8 Advanced X-Ray Diffractometer with Cu Kα radiation (*λ* = 0.154 nm). The surface charges of all the samples were measured by Zeta Potential Analyzer (ZetaPALS, Brookhaven Instruments, USA). The photocatalysis degradation was performed by using 300 W Xe lamp (PLS-SXE300D/300DUV, Beijing perfect light technology co. LTD) as the light source using a cut-off filter of 420 nm to remove UV light.

### Photocatalysis test

The photo-assisted catalytic degradation of methylene blue (MB) was carried out in a 100 mL quartz reactor with 15 mg of as-synthesized sample, 0.5 mM peroxymonosulfate (PMS) and 50 mL of MB solution (100 ppm, pH = 7). The suspension was stirred in dark for 60 min to establish the adsorption equilibrium before irradiation and addition of PMS. Then, 300 W Xe lamp was used as the source of visible light using a cut-off filter of 420 nm to remove UV light. The catalysis sample was collected from the reactor and filtered (0.22 μm) to remove the particles with adding methanol to quench the radicals. The concentrations of MB were monitored by measuring the absorption intensity at maximum absorbance wavelength (*λ* = 660 nm) using a UV-vis spectrophotometer. The photocatalytic performances of all samples were compared based on the same dosages.

The photocurrents were measured using 0.1 M Na_2_SO_4_ as electrolyte on a CHI 760 E electrochemical workstation with a three-electrode cell system including a Ag/AgCl electrode as reference electrode, MOF/ITO as working electrode and Pt wire as counter electrode. 300 W Xe lamp was used as the source of visible light using a cut-off filter of 420 nm to remove UV light.

## Supplementary information

Supplementary Information

## Data Availability

The data that support the plots within this paper and other findings of this study are available from the corresponding authors upon reasonable request.

## References

[1] Liu X (2018). Complex silica composite nanomaterials templated with DNA origami. Nature.

[2] Xiang R (2020). One-dimensional Van der Waals heterostructures. Science.

[3] Geim AK, Grigorieva IV (2013). Van der Waals heterostructures. Nature.

[4] Joshi RK, Schneider JJ (2012). Assembly of one dimensional inorganic nanostructures into functional 2D and 3D architectures. Synthesis, arrangement and functionality. Chem. Soc. Rev..

[5] Yao YG (2018). Carbothermal shock synthesis of high-entropy-alloy nanoparticles. Science.

[6] Ha MJ (2019). Multicomponent plasmonic nanoparticles: from heterostructured nanoparticles to colloidal composite nanostructures. Chem. Rev..

[7] Wang JY, Wan JW, Yang NL, Li Q, Wang D (2020). Hollow multishell structures exercise temporal-spatial ordering and dynamic smart behavior. Nat. Rev. Chem..

[8] Zhou L (2017). Intricate hollow structures: controlled synthesis and applications in energy storage and conversion. Adv. Mater..

[9] Yu L, Hu H, Wu HB, Lou XW (2017). Complex hollow nanostructures: synthesis and energy-related applications. Adv. Mater..

[10] Wu XJ (2016). Controlled growth of high-density CdS and CdSe nanorod arrays on selective facets of two-dimensional semiconductor nanoplates. Nat. Chem..

[11] Wang WX (2020). Engine-trailer-structured nanotrucks for efficient nano-bio interactions and bioimaging-guided drug delivery. Chem.

[12] Zhao TC (2019). Surface-kinetics mediated mesoporous multipods for enhanced bacterial adhesion and inhibition. Nat. Commun..

[13] Yaghi OM (2003). Reticular synthesis and the design of new materials. Nature.

[14] Lee JY (2009). Metal-organic framework materials as catalysts. Chem. Soc. Rev..

[15] Xiao JD, Jiang HL (2019). Metal-organic frameworks for photocatalysis and photothermal catalysis. Acc. Chem. Res..

[16] Li JR, Sculley J, Zhou HC (2012). Metal-organic frameworks for separations. Chem. Rev..

[17] Cai DP (2016). Rational synthesis of metal-organic framework composites, hollow structures and their derived porous mixed metal oxide hollow structures. J. Mater. Chem. A.

[18] Yang HZ, Wang X (2019). Secondary-component incorporated hollow mofs and derivatives for catalytic and energy-related applications. Adv. Mater..

[19] Guan BY, Yu XY, Wu HB, Lou XW (2017). Complex nanostructures from materials based on metal-organic frameworks for electrochemical energy storage and conversion. Adv. Mater..

[20] Liu C (2020). Amorphous metal-organic framework-dominated nanocomposites with both compositional and structural heterogeneity for oxygen evolution. Angew. Chem. Int. Ed..

[21] Zhao MT (2016). Metal-organic frameworks as selectivity regulators for hydrogenation reactions. Nature.

[22] Yang QH, Xu Q, Jiang HL (2017). Metal-organic frameworks meet metal nanoparticles: synergistic effect for enhanced catalysis. Chem. Soc. Rev..

[23] Chen WH, Vázquez-González M, Zoabi A, Rezip RA, Willner I (2018). Biocatalytic cascades driven by enzymes encapsulated in metal-organic framework nanoparticles. Nat. Catal..

[24] Tang J (2015). Thermal conversion of core-shell metal-organic frameworks: a new method for selectively functionalized nanoporous hybrid carbon. J. Am. Chem. Soc..

[25] Yang J (2015). Hollow Zn/Co ZIF particles derived from core-shell ZIF-67@ZIF-8 as selective catalyst for the semi-hydrogenation of acetylene. Angew. Chem. Int. Ed..

[26] Furukawa S (2009). Heterogeneously hybridized porous coordination polymer crystals: fabrication of heterometallic core-shell single crystals with an in-plane rotational epitaxial relationship. Angew. Chem. Int. Ed..

[27] Yang X (2018). One-step synthesis of hybrid core-shell metal-organic frameworks. Angew. Chem. Int. Ed..

[28] Choi S, Kim T, Ji H, Lee HJ, Oh M (2016). Isotropic and anisotropic growth of metal-organic framework (MOF) on MOF: logical inference on MOF structure based on growth behavior and morphological feature. J. Am. Chem. Soc..

[29] Luo TY (2019). Multivariate stratified metal-organic frameworks: diversification using domain building blocks. J. Am. Chem. Soc..

[30] Mutruc D (2019). Modulating guest uptake in core-shell MOFs with visible light. Angew. Chem. Int. Ed..

[31] Li T, Sullivan JE, Rosi NL (2017). Design and preparation of a core-shell metal-organic framework for selective CO_2_ capture. J. Am. Chem. Soc..

[32] Yao MS (2019). Van der Waals heterostructured MOF-on-MOF thin films: cascading functionality to realize advanced chemiresistive sensing. Angew. Chem. Int. Ed..

[33] Guan BY, Yu L, Lou XW (2016). A dual-metal-organic-framework derived electrocatalyst for oxygen reduction. Energy Environ. Sci..

[34] Pambudi FI, Anderson MW, Attfield MP (2019). Unveiling the mechanism of lattice-mismatched crystal growth of a core-shell metal-organic framework. Chem. Sci..

[35] Lee S, Oh S, Oh M (2020). Atypical hybrid metal-organic frameworks (MOFs): a combinative process for MOF‐on‐MOF growth, etching, and structure transformation. Angew. Chem. Int. Ed..

[36] McDonald KA, Feldblyum JI, Koh K, Wong-Foy AG, Matzger AJ (2015). Polymer@ MOF@ MOF:“Grafting from” atom transfer radical polymerization for the synthesis of hybrid porous solids. Chem. Commun..

[37] Faustini M (2013). Microfluidic approach toward continuous and ultrafast synthesis of metal–organic framework crystals and hetero structures in confined microdroplets. J. Am. Chem. Soc..

[38] Ji H (2018). Improvement in crystallinity and porosity of poorly crystalline metal–organic frameworks (MOFs) through their induced growth on a well-crystalline MOF template. Inorg. Chem..

[39] Lee HJ, Cho YJ, Cho W, Oh M (2013). Controlled isotropic or anisotropic nanoscale growth of coordination polymers: formation of hybrid coordination polymer particles. ACS Nano.

[40] Wang Z (2014). Nanoporous designer solids with huge lattice constant gradients: multiheteroepitaxy of metal–organic frameworks. Nano Lett..

[41] Gu YF (2017). Controllable modular growth of hierarchical MOF-on-MOF Architectures. Angew. Chem. Int. Ed..

[42] Ikigaki K (2019). MOF-on-MOF: oriented growth of multiple layered thin films of metal-organic frameworks. Angew. Chem. Int. Ed..

[43] Lee G, Lee S, Oh S, Kim D, Oh M (2020). Tip-to-middle anisotropic MOF-On-MOF growth with a structural adjustment. J. Am. Chem. Soc..

[44] Hardi MD (2009). A new photoactive crystalline highly porous titanium (IV) dicarboxylate. J. Am. Chem. Soc..

[45] Park KS (2006). Exceptional chemical and thermal stability of zeolitic imidazolate frameworks. Proc. Natl Acad. Sci. USA.

[46] Liu C (2020). Site-specific growth of MOF-on-MOF heterostructures with controllable nano-architectures: beyond the combination of MOF analogues. Chem. Sci..

[47] Chen YZ (2015). From bimetallic metal-organic framework to porous carbon: high surface area and multicomponent active dopants for excellent electrocatalysis. Adv. Mater..

[48] Guo F (2019). Facet-dependent photocatalytic hydrogen production of metal–organic framework NH_2_-MIL-125(Ti). Chem. Sci..

[49] Chen F (2020). Catalytic degradation of ciprofloxacin by a visible-light-assisted peroxymonosulfate activation system: performance and mechanism. Water Res..

[50] Anipsitakis GP, Dionysiou DD (2004). Radical generation by the interaction of transition metals with common oxidants. Environ. Sci. Technol..

[51] Li P (2019). Metal-organic frameworks with photocatalytic bactericidal activity for integrated air cleaning. Nat. Commun..

