# Supplementary material for: Ternary MOF-on-MOF heterostructures with controllable architectural and compositional complexity via multiple selective assembly
Source: Nat Commun. 2020 Oct 2;11:4971. doi: 10.1038/s41467-020-18776-z (PMC7532534; doi:10.1038/s41467-020-18776-z)
Supplement: Supplementary file 1 — Supplementary Information [file 41467_2020_18776_MOESM1_ESM.pdf]

Supplementary information

Ternary MOF-on-MOF heterostructures with controllable architectural and compositional complexity via multiple selective assembly

Liu *et al.*

Supplementary Figures

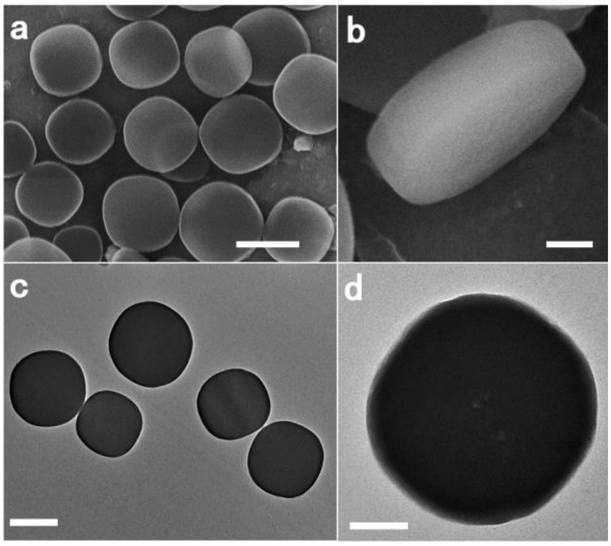

Supplementary Figure 1. (a, b) SEM and (c, d) TEM images of MIL-125. Scale bars are 500 nm (a, c), 100 nm (b) and 200 nm (d).

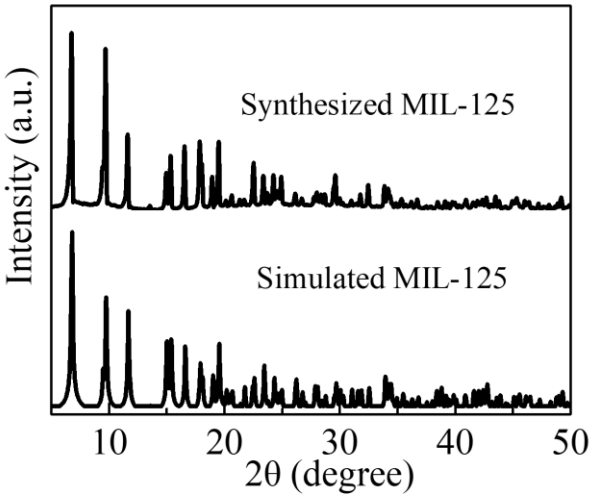

Supplementary Figure 2. XRD patterns of MIL-125.

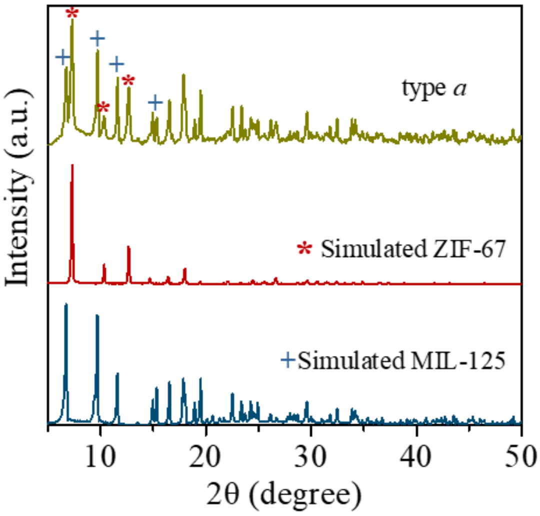

Supplementary Figure 3. XRD patterns of type *a* MIL-125@ZIF-67 heterostructure.

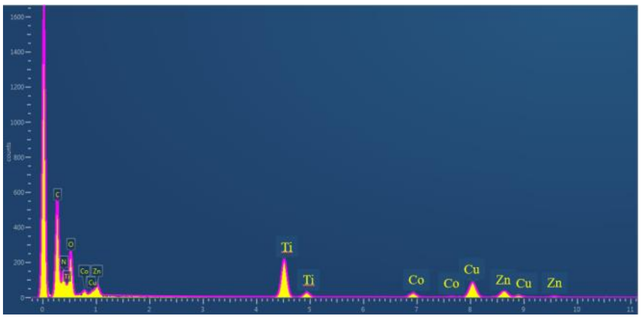

Supplementary Figure 4. EDX spectrum of type *A* heterostructure.

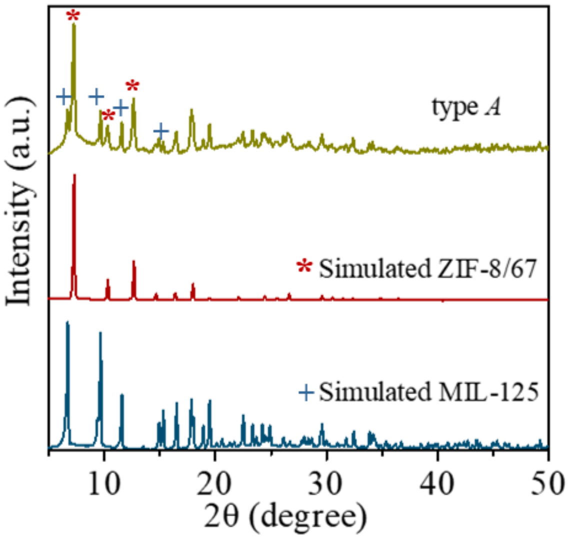

Supplementary Figure 5. XRD patterns of type *A* MIL-125@ZIF-67@ZIF-8 heterostructure.

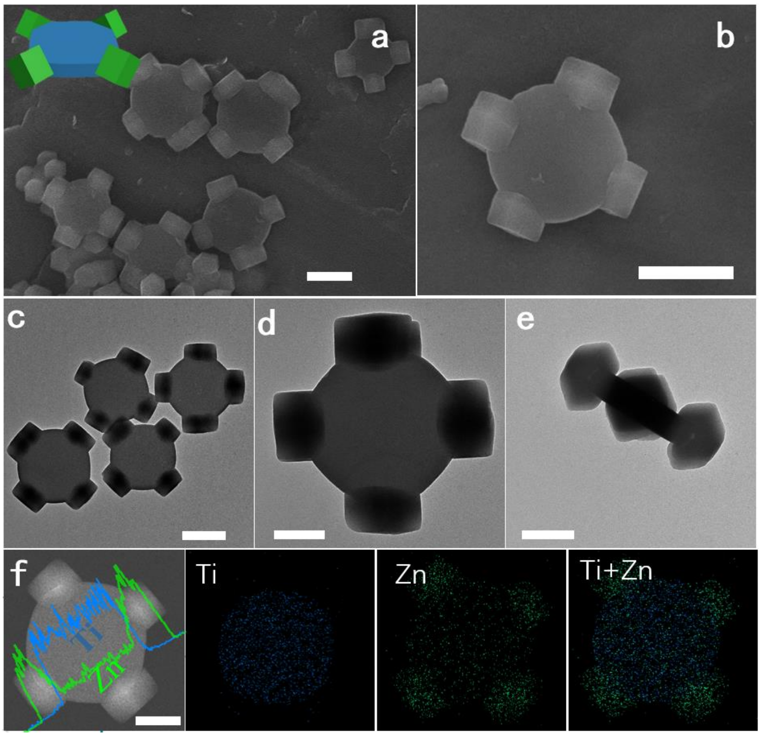

Supplementary Figure 6. (a, b) SEM images, (c-e) TEM images, (f) Scanning transmission electron microscopy image (STEM) and corresponding element mapping images of type *b* MIL-125@ZIF-8 heterostructure. Scale bars are 500 nm (a-c) and 200 nm (d-f).

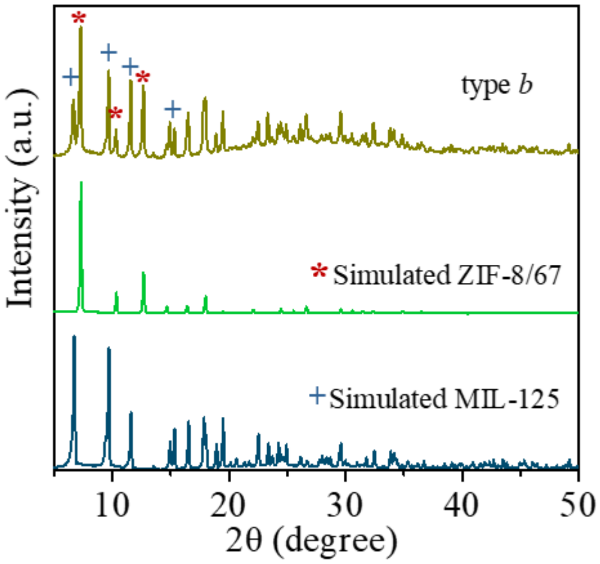

Supplementary Figure 7. XRD patterns of type *b* MIL-125@ZIF-8 heterostructure.

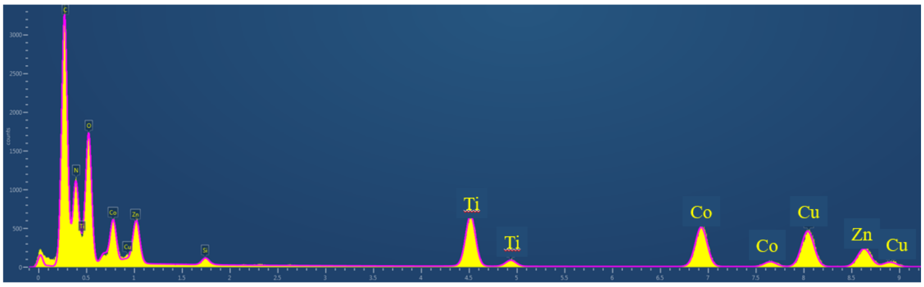

Supplementary Figure 8. EDX spectrum of type *B* heterostructure.

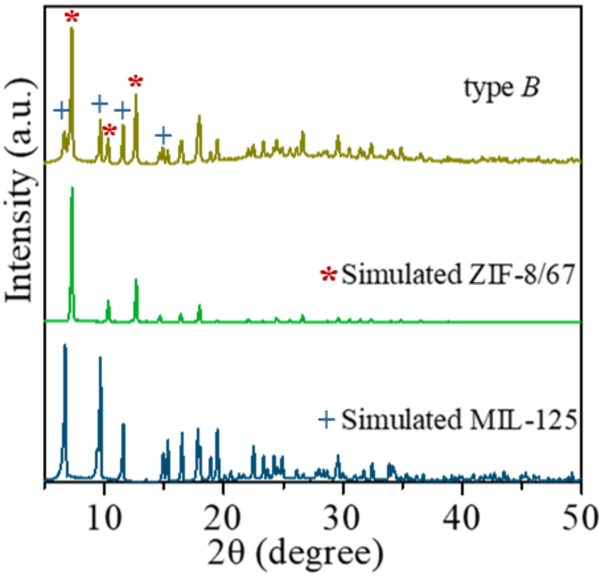

Supplementary Figure 9. XRD patterns of type *B* MIL-125@ZIF-8@ZIF-67 heterostructure.

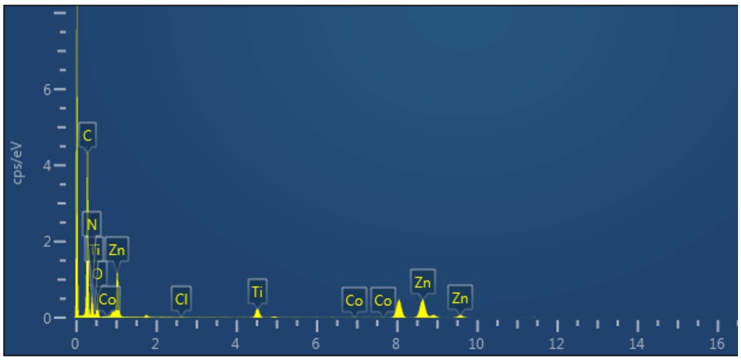

Supplementary Figure 10. EDX spectrum of type *C* heterostructure.

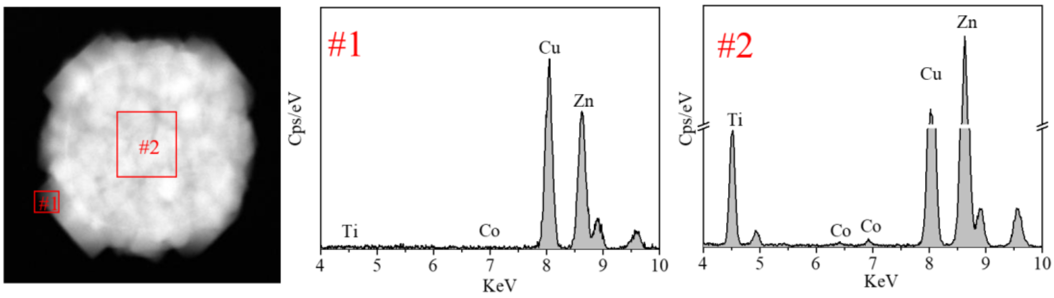

Supplementary Figure 11. STEM image and spot scan spectra of type *C* heterostructure.

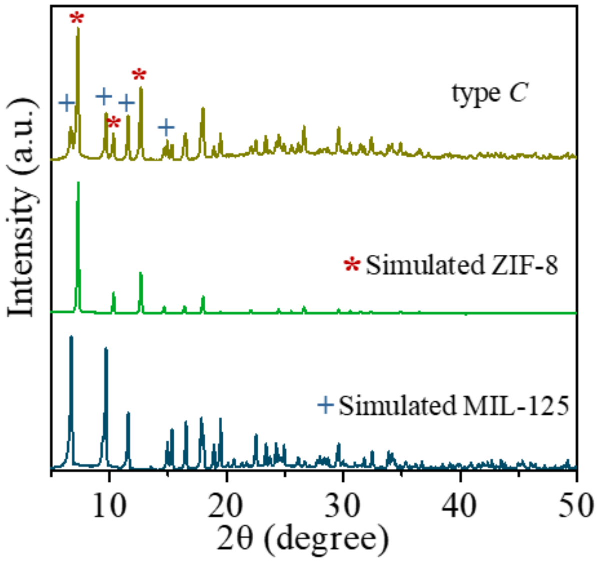

Supplementary Figure 12. XRD patterns of type *C* MIL-125@ZIF-8/ Zn, Co-ZIF heterostructure.

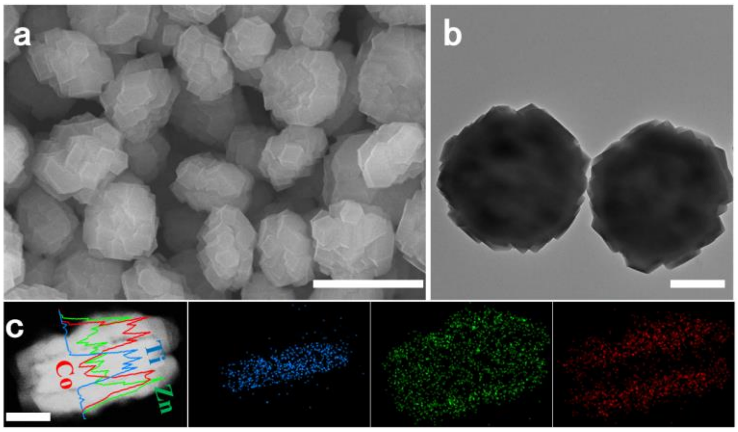

Supplementary Figure 13. (a) SEM image, (b) TEM image, (c) STEM image together

with line scanning spectra and element mapping images of type *A*-1 heterostructure. The scale bars are 1 μm (a), 500 nm (b) and 200 nm (c).

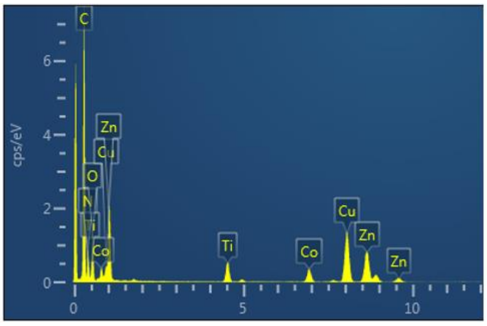

Supplementary Figure 14. EDX spectrum of type *A*-1 heterostructure.

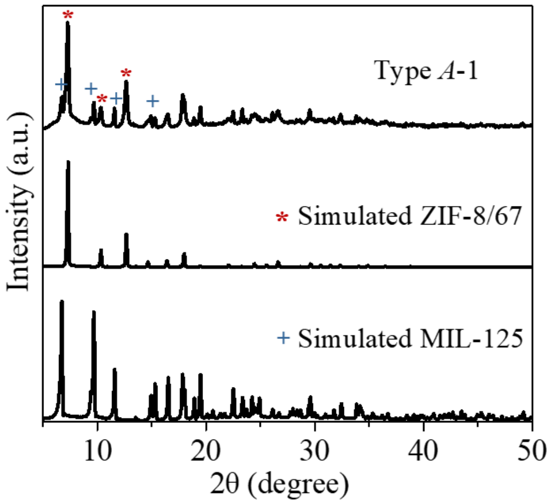

Supplementary Figure 15. XRD patterns of type *A*-1 heterostructure.

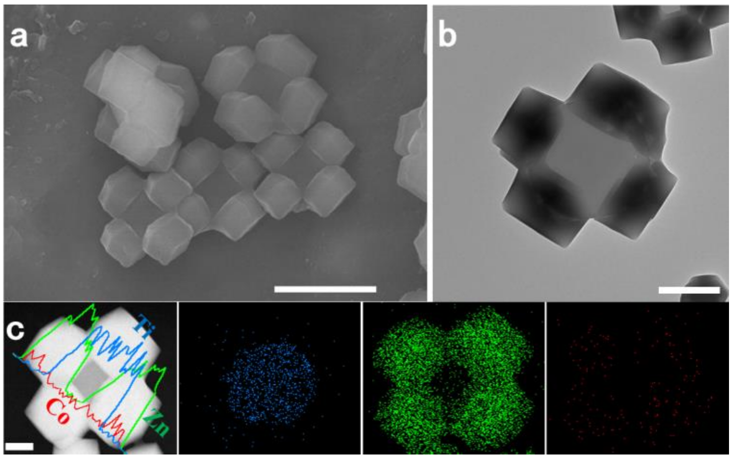

Supplementary Figure 16. (a) SEM image, (b) TEM image, (c) STEM image together with line scanning spectra and element mapping images of type *B*-1 heterostructure. The scale bars are 1 μm (a), 500 nm (b) and 200 nm (c).

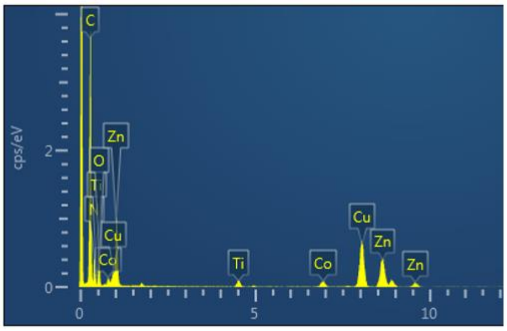

Supplementary Figure 17. EDX spectrum of type *B*-1 heterostructure.

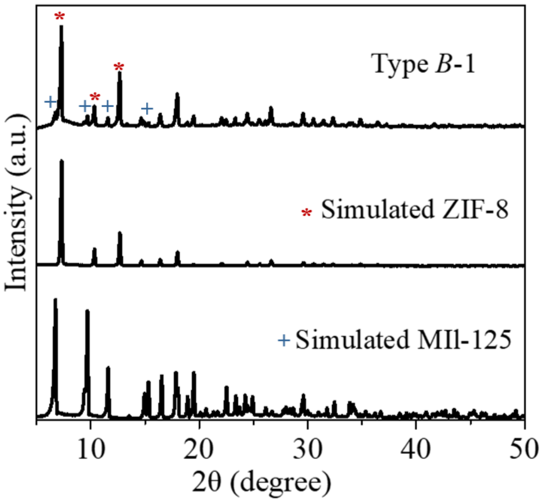

Supplementary Figure 18. XRD patterns of type *B*-1 heterostructure.

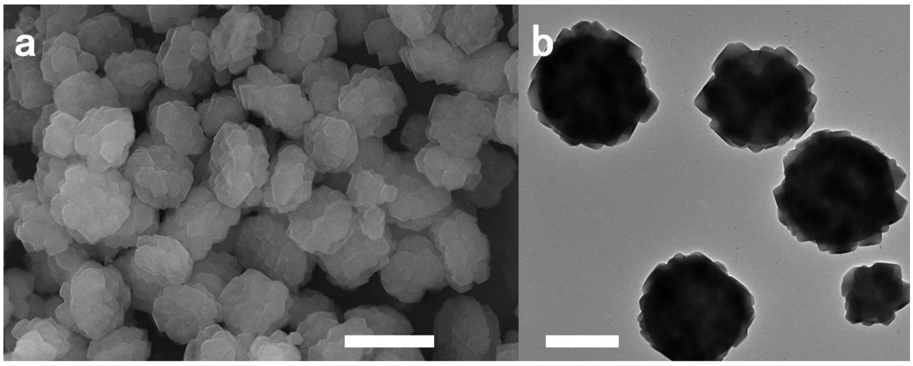

Supplementary Figure 19. SEM and TEM images of type *A* MIL-125@ZIF-67@ZIF-8 heterostructure after sonication in methanol with frequency of 50 KHz for 5 min. The scale bars are 1 μm and 500 nm respectively.

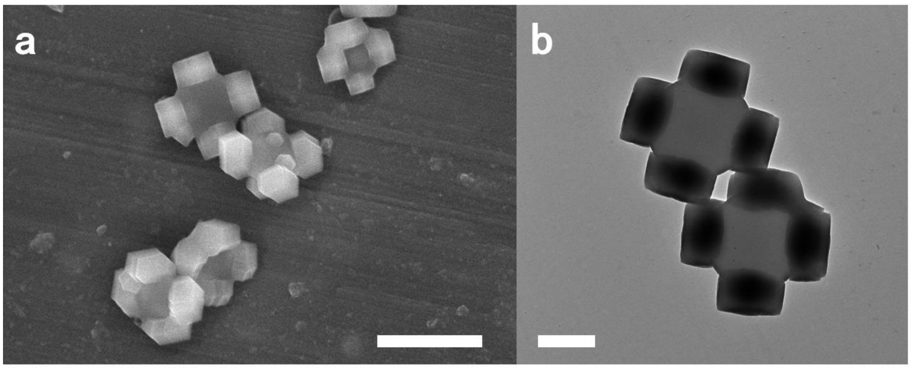

Supplementary Figure 20. SEM and TEM images of type *B* MIL-125@ZIF-8@ZIF-67 heterostructure after sonication in methanol with frequency of 50 KHz for 5 min. The scale bars are 1 μm and 500 nm respectively.

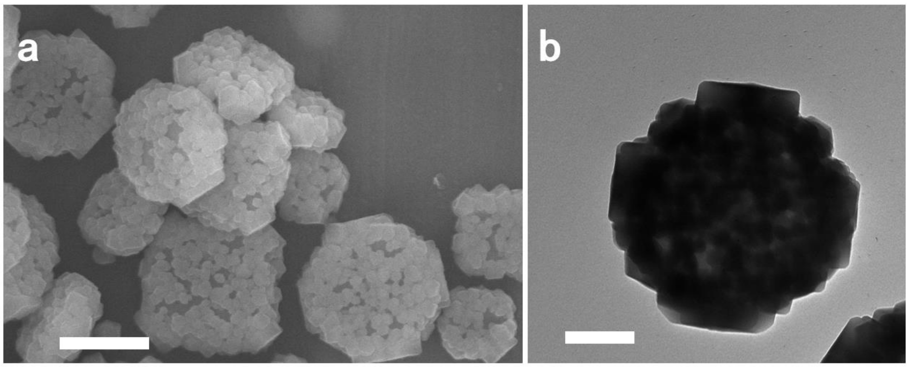

Supplementary Figure 21. SEM and TEM images of type *C* MIL-125@ZIF-8/Zn, Co-ZIF heterostructure after sonication in methanol with frequency of 50 KHz for 5 min. The scale bars are 500 nm and 200 nm respectively.

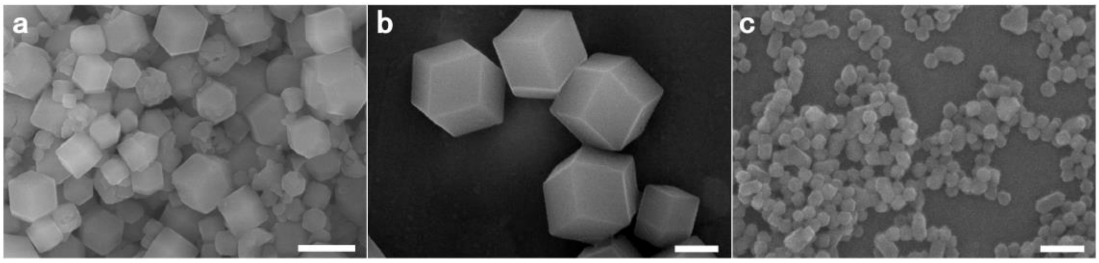

Supplementary Figure 22. SEM images of (a) ZIF-67, (b) ZIF-8 and (c) Zn, Co-ZIF.

The scale bars are 1 μm (a) and 500 nm (b, c).

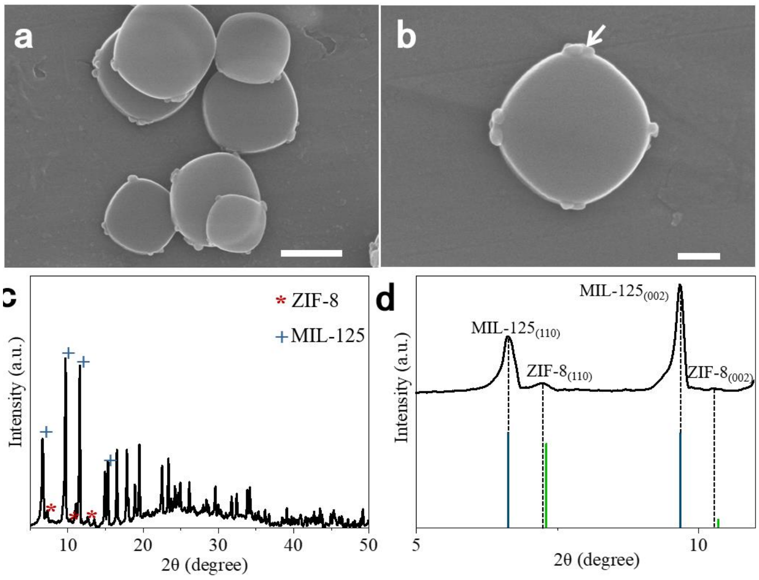

Supplementary Figure 23. (a, b) SEM images (c, d) XRD patterns of sample after growth of ZIF-8 on MIL-125 for 2 min (denoted as MIL-125@ZIF-8-2 min). The scale bars are 500 nm (a) and 200 nm (b).

Figure S23 a and b are SEM images taken from initial growth of ZIF-8 on the MIL-125 host (products collected after 2 min of ZIF-8 growth). Some nucleates are seen only at the corner of the host MIL-125, rather than on the side or top surface, indicating that only {110} surfaces of MIL-125 are the nucleation sites for growing ZIF-8.

The diffraction peaks indexed to ZIF-8 are also found in the XRD pattern of MIL-125@ZIF-8-2 min (Figure S23c), consistent with the existence of ZIF-8 nucleates. Figure S23d shows an enlarged XRD pattern, in which the observed diffraction peaks are compared with the standard diffraction peaks. The diffraction peaks attributed to MIL-125 fit well with the standard ones. In contrast, the diffraction peaks attributed to ZIF-8 (110 and 002) have slightly shifted to a smaller angle when compared with the

standard peaks, showing a lattice expansion of ~0.95% for 110 diffraction for ZIF-8 nucleates grown on MIL-125. The structural deformation reduces the lattice mismatch between ZIF-8 and MIL-125, suggesting the epitaxial growth of ZIF-8 on MIL-125.[S1]

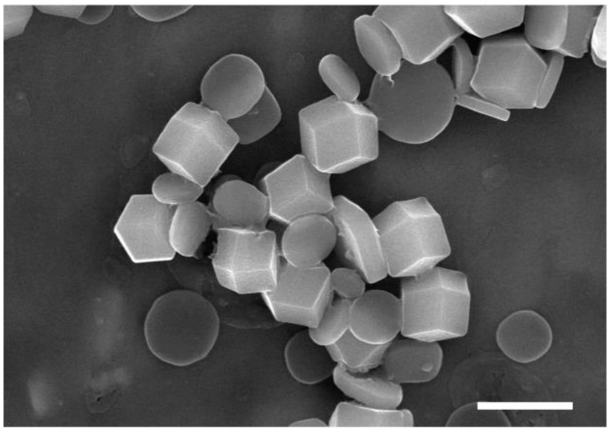

Supplementary Figure 24. SEM image of sample by adding MIL-125 after growth of ZIF-8 for 3 min for pre-nucleation. The scale bar is 1 μm.

MIL-125 were added after homogenous nucleation and growth of ZIF-8 for 5 min, leading to the mixture of dodecahedral ZIF-8 particles and MIL-125 (Figure S24). This result indicates the growth of MIL-125@ZIF-8 is through heterogeneous nucleation of ZIF-8 clusters on {110} facets of MIL-125.

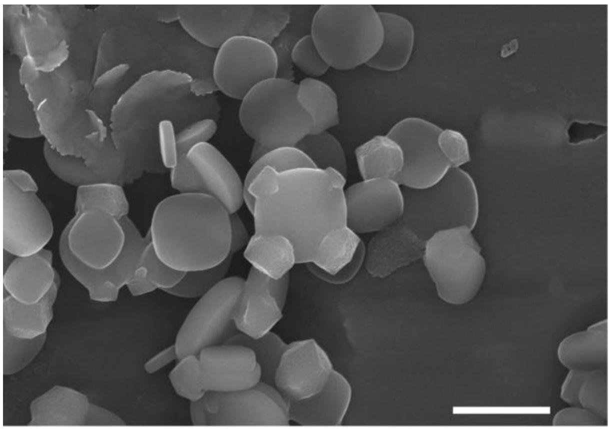

Supplementary Figure 25. SEM image of sample obtained by adding Zn^{2+} first and then 2-MeIM. The scale bar is 1 μm.

By adding Zn^{2+} first and then 2-MeIM to react with MIL-125, an inhomogeneous structure with partial MIL-125 anchored by ZIF-8 was formed (Figure S25), different from type *a* MIL-125@ZIF-8 heterostructure. It is reported that the {110} facets of MIL-125 have high content of exposed Ti-O clusters.[S2] Our result suggests that the interaction between 2-MeIM and Ti-O clusters exposed on {110} facets of MIL-125 is also associated with the site-selective growth of MIL-125@ZIF-8.

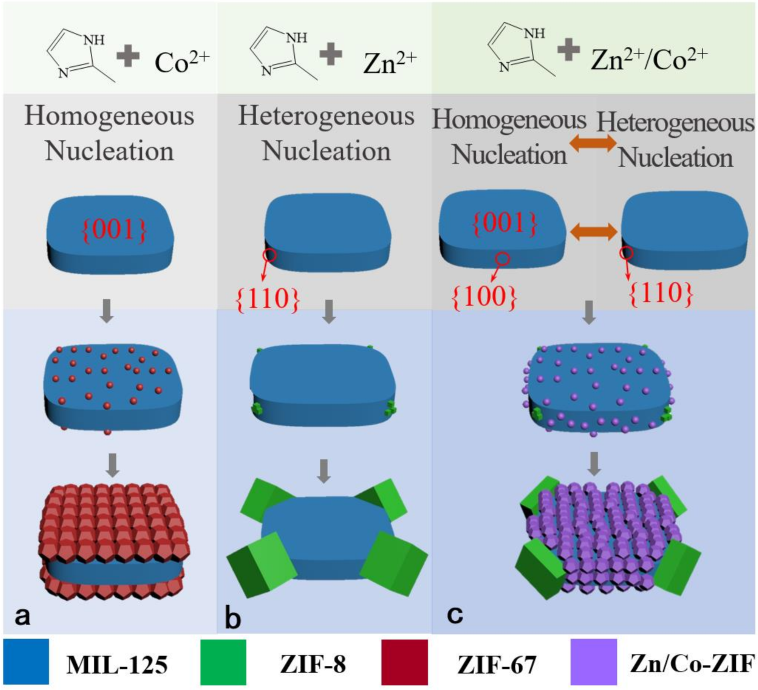

Supplementary Figure 26. The nucleation and growth manners of ZIFs with MIL-125 as host.

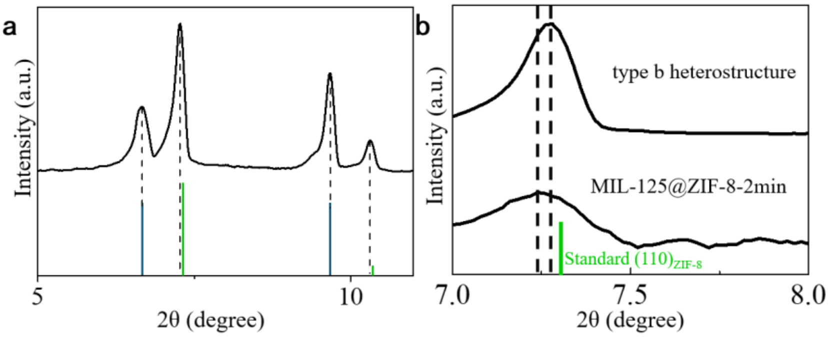

Supplementary Figure 27. The enlarged XRD patterns of (a) type *b* in the range of 2θ from 5°-12°, (b, c) type *b* and MIL-125@ZIF-8-2 min.

As shown in Figure S27, after growth of ZIF-8 for 2 h, the lattice expansion of ZIF-8 in type *b* binary heterostructure decrease from ~0.95% (MIL-125@ZIF-8-2 min) to 0.57% calculated from the 110 diffraction, consistent with the further growth of ZIF-8.

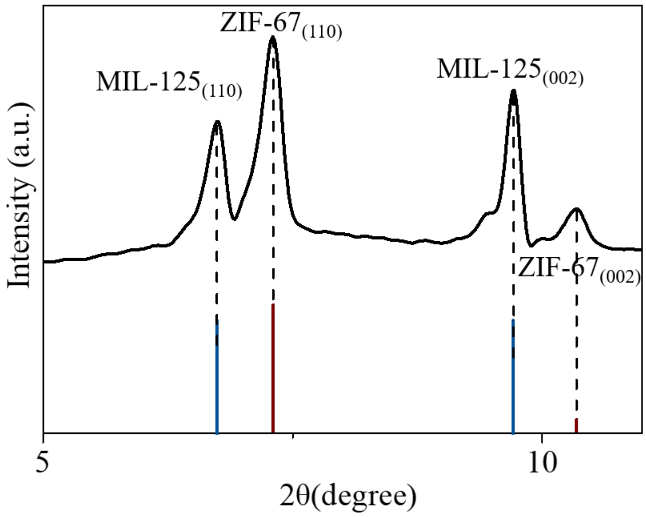

Supplementary Figure 28. The enlarged XRD pattern of type *a* in the range of 2θ from 5°-12°.

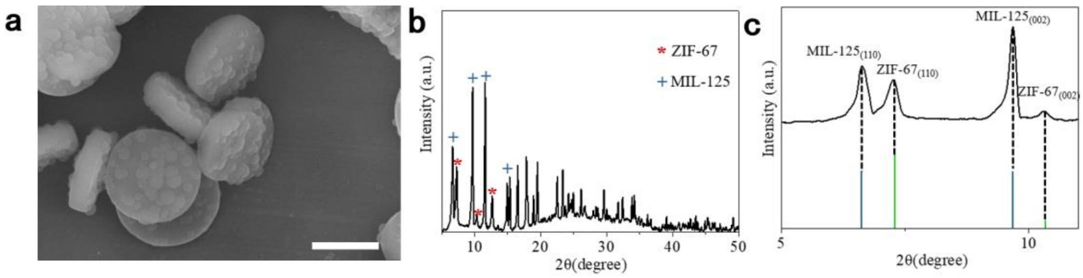

Supplementary Figure 29. (a) SEM images and (b, c) XRD patterns of sample after growth of ZIF-67 on MIL-125 for 2 min. The scale bar is 500 nm.

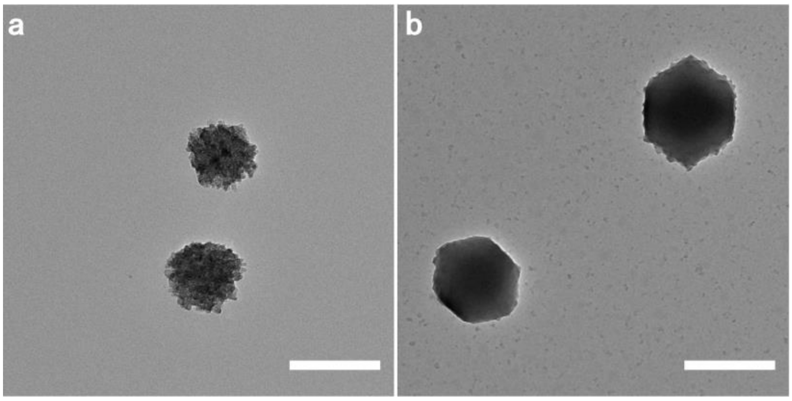

Supplementary Figure 30. TEM images of (a) ZIF-8 and (b) ZIF-67 by growth for 5 min. The scale bars are 100 nm and 200 nm, respectively.

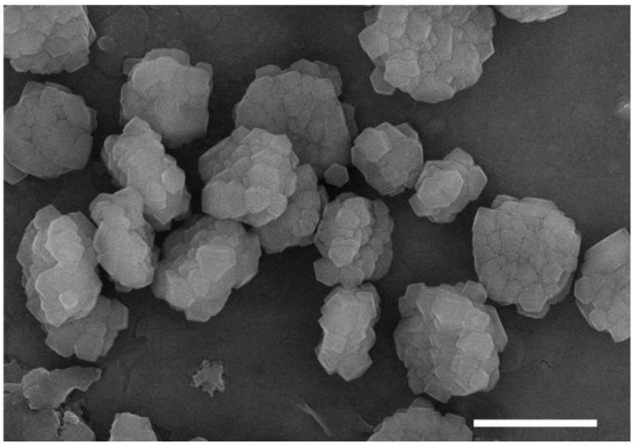

Supplementary Figure 31. SEM image of sample obtained by adding Co^{2+} first and then 2-MeIM. The scale bar is 1 μm.

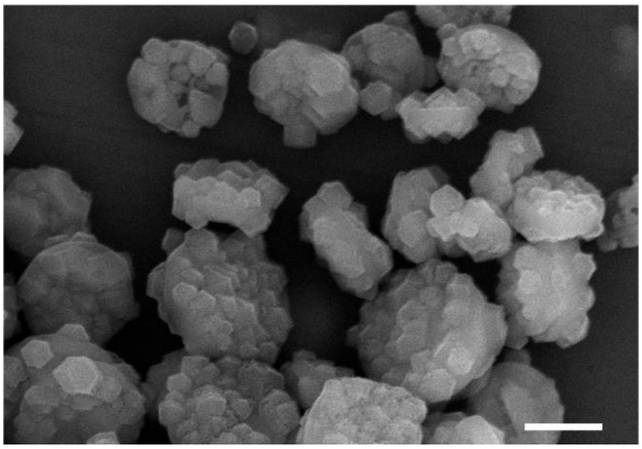

Supplementary Figure 32. SEM image of sample by adding MIL-125 after growth of ZIF-67 for 3 min for pre-nucleation. The scale bar is 500 nm.

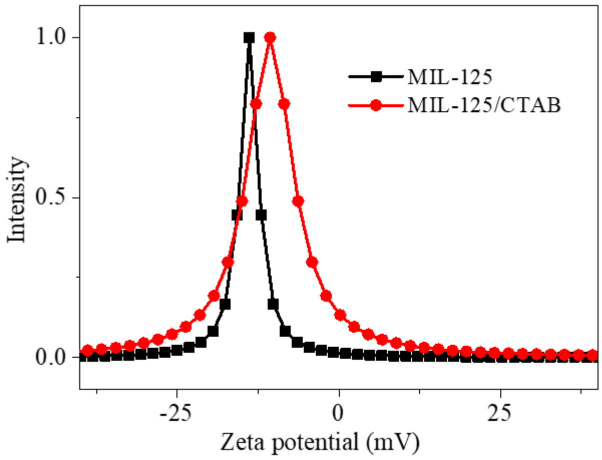

Supplementary Figure 33. Zeta potential of MIL-125 with and without CTAB.

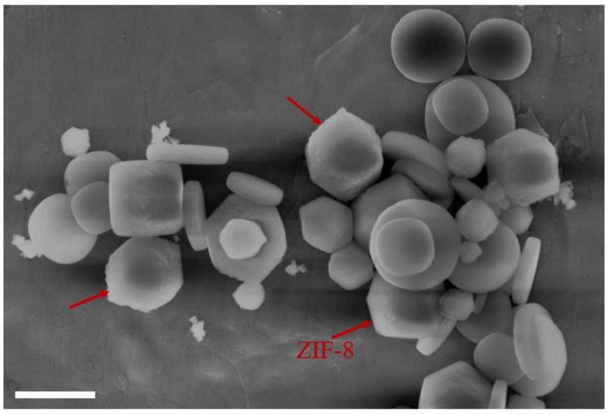

Supplementary Figure 34. SEM image of sample by growth of ZIF-8 with MIL-125 as host and addition of CTAB. The scale bar is 1 μm.

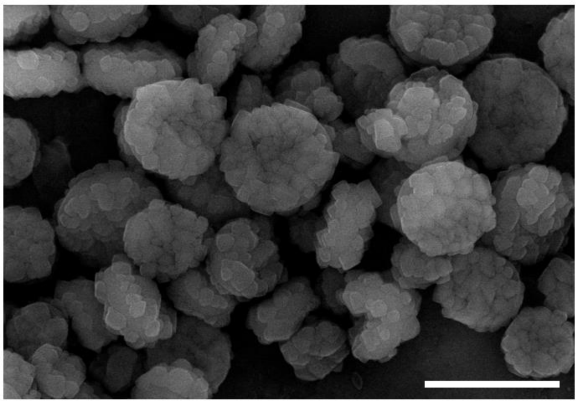

Supplementary Figure 35. SEM image of sample by growth of ZIF-67 with MIL-125 as host and addition of CTAB. The scale bar is 1 μm.

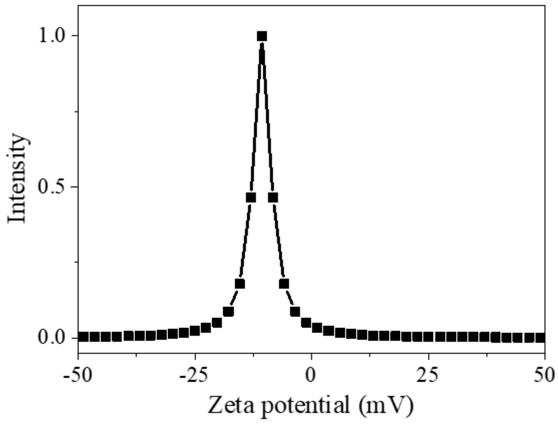

Supplementary Figure 36. Zeta potential of ZIF-67.

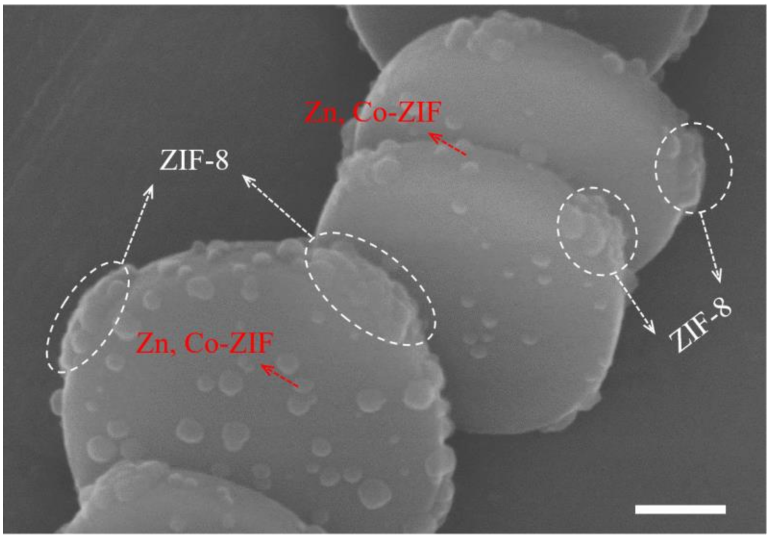

Supplementary Figure 37. SEM images of sample after reaction of Zn^{2+}, Co^{2+} and 2-MeIM with MIL-125 for 2 min. The scale bar is 200 nm.

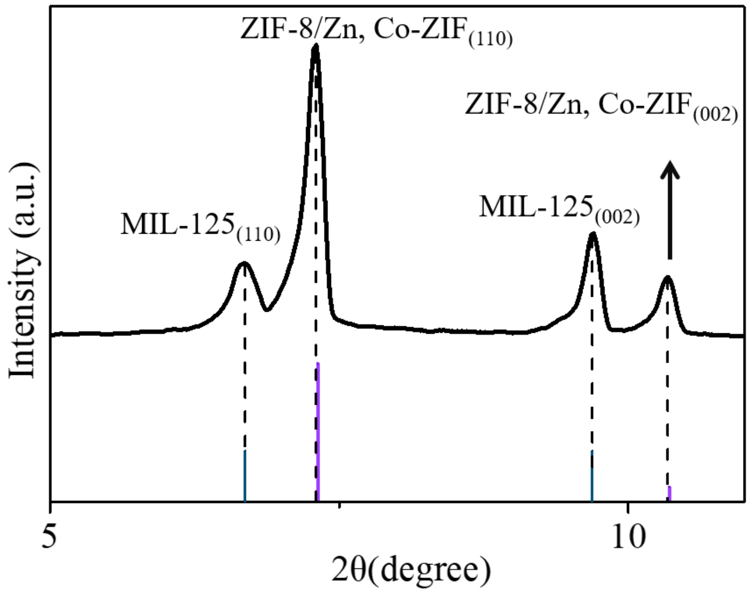

Supplementary Figure 38. The enlarged XRD pattern of type *C* in the range of 2θ from 5°-12°.

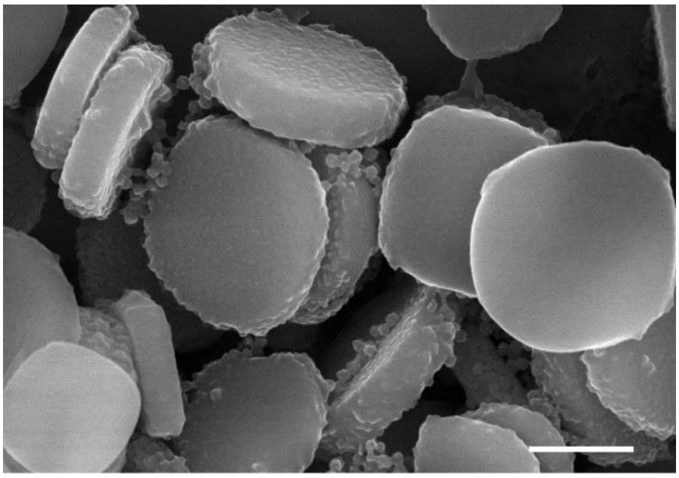

Supplementary Figure 39. SEM image of sample by adding MIL-125 after reaction of Zn^{2+}, Co^{2+} and 2-MeIM for 3 min for pre-nucleation. The scale bar is 500 nm.

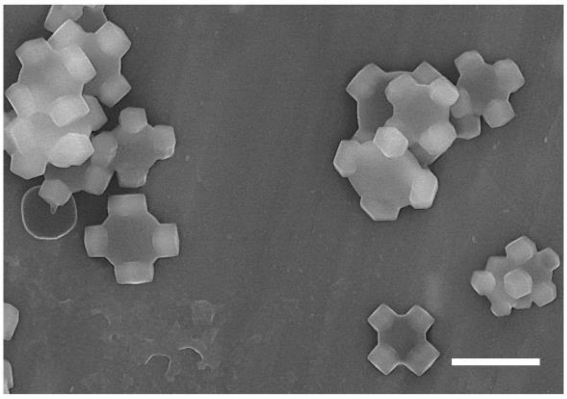

Supplementary Figure 40. SEM image of sample by reaction of Zn^{2+}, Co^{2+} (Zn/Co molar ratio of 8.5:1.5) and 2-MeIM with MIL-125.

Figure S39 shows the SEM image of sample by using Zn/Co molar ratio of 8.5:1.5. The resultant structure is similar with type *b* binary hybrid, different from type *C* (Zn/Co molar ratio of 7.5:2.5).

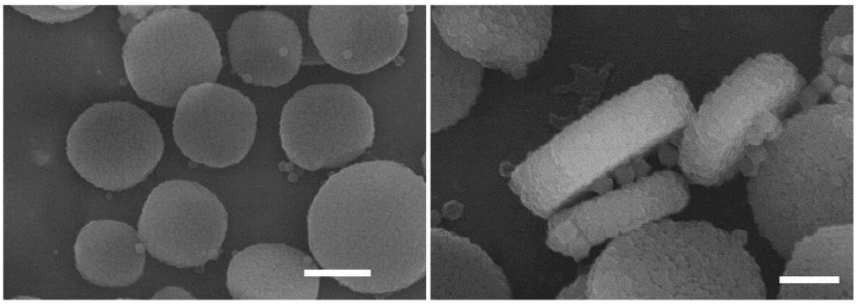

Supplementary Figure 41. SEM image of sample by reaction of Zn^{2+}, Co^{2+} (Zn/Co molar ratio of 0.5:0.5) and 2-MeIM with MIL-125.

When changing the Zn/Co molar ratio to 5:5, the MIL-125 is fully coated by small crystals without growth of larger ZIF-8 on the four corners (Figure S40), generating a core-shell structure. The results of Figure S22 and S23 verify the optimized Zn/Co ratio is ~7.5/2.5, which enables the co-existence of two competitive growth mechanisms and thus the formation of type *C* ternary MOF-on-MOF heterostructure.

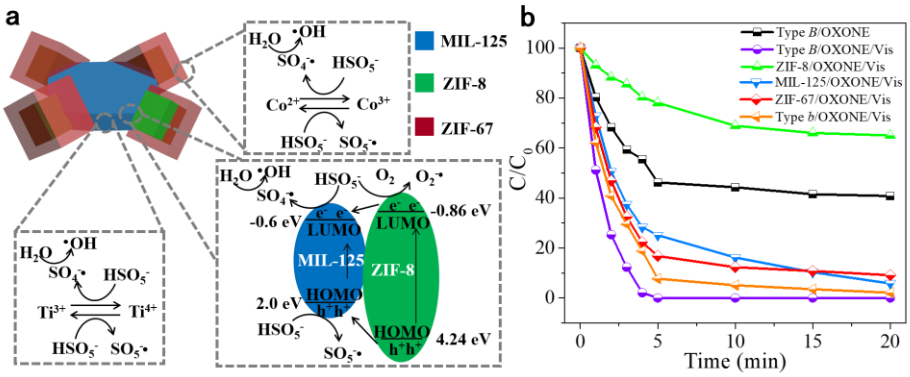

Supplementary Figure 42. (a) Illustration of the photo-assisted catalytic degradation process towards MB degradation using type *B* heterostructure. (b) Degradation efficiency of type *B* in the dark, and type *B*, ZIF-8, ZIF-67, MIL-125, type *b* under irradiation. HOMO and LUMO represent highest occupied molecular orbital and lowest unoccupied molecular orbital respectively.

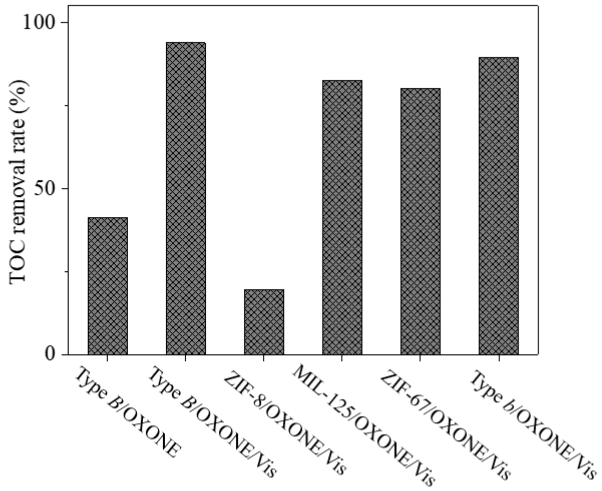

Supplementary Figure 43. TOC removal efficiency of type *B* in the dark, and type *B*, ZIF-8, ZIF-67, MIL-125, type *b* under irradiation.

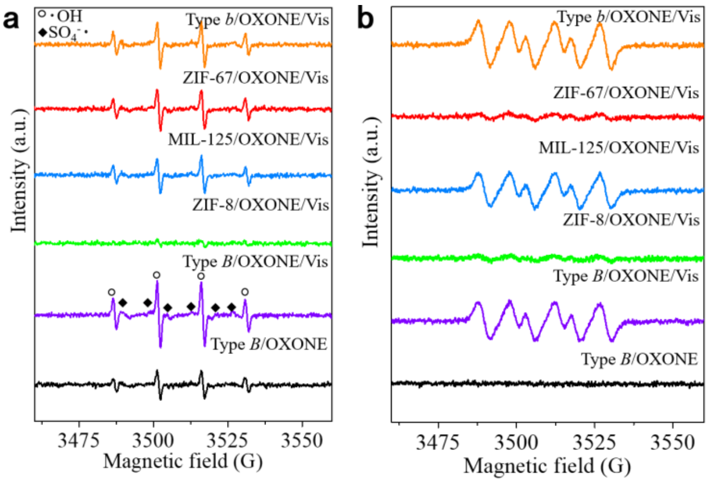

Supplementary Figure 44. ESR signals of (a) DMPO-•OH and DMPO-SO_4•$^-$ and (b) DMPO- O_2•$^-$ in various catalytic systems.

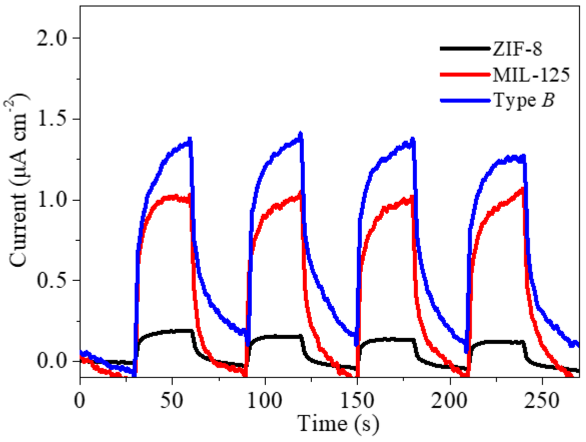

Supplementary Figure 45. Transient photocurrent of ZIF-8, MIL-125 and type *B* heterostructure with light on/off cycles under visible light irradiation.

Supplementary Reference

(1) Liu, C. et al. Site-Specific Growth of MOF-on-MOF Heterostructures with Controllable Nano-Architectures: Beyond the Combination of MOF Analogues. *Chem. Sci.* **11**, 3680 (2020).

(2) Guo, F. et al. Facet-dependent photocatalytic hydrogen production of metal–organic framework NH_2-MIL-125(Ti). *Chem. Sci,* **10**, 4834 (2019).
